# Prevalence and distribution of extended-spectrum β-lactamase and AmpC-producing *Escherichia coli* in two New Zealand dairy farm environments

**DOI:** 10.3389/fmicb.2022.960748

**Published:** 2022-08-11

**Authors:** Rose M. Collis, Patrick J. Biggs, Sara A. Burgess, Anne C. Midwinter, Gale Brightwell, Adrian L. Cookson

**Affiliations:** ^1^The Hopkirk Research Institute, AgResearch Ltd., Massey University, Palmerston North, New Zealand; ^2^^m^EpiLab, School of Veterinary Science, Massey University, Palmerston North, New Zealand; ^3^School of Natural Sciences, Massey University, Palmerston North, New Zealand; ^4^New Zealand Food Safety Science and Research Centre, Massey University, Palmerston North, New Zealand

**Keywords:** *Escherichia coli*, antimicrobial resistance, AmpC, ESBL, dairy, third-generation cephalosporin resistance, genomics

## Abstract

Antimicrobial resistance (AMR) is a global threat to human and animal health, with the misuse and overuse of antimicrobials being suggested as the main driver of resistance. In a global context, New Zealand (NZ) is a relatively low user of antimicrobials in animal production. However, the role antimicrobial usage on pasture-based dairy farms, such as those in NZ, plays in driving the spread of AMR within the dairy farm environment remains equivocal. Culture-based methods were used to determine the prevalence and distribution of extended-spectrum β-lactamase (ESBL)- and AmpC-producing *Escherichia coli* from farm environmental samples collected over a 15-month period from two NZ dairy farms with contrasting management practices. Whole genome sequencing was utilised to understand the genomic epidemiology and antimicrobial resistance gene repertoire of a subset of third-generation cephalosporin resistant *E. coli* isolated in this study. There was a low sample level prevalence of ESBL-producing *E. coli* (faeces 1.7%; farm dairy effluent, 6.7% from Dairy 4 and none from Dairy 1) but AmpC-producing *E. coli* were more frequently isolated across both farms (faeces 3.3% and 8.3%; farm dairy effluent 38.4%, 6.7% from Dairy 1 and Dairy 4, respectively). ESBL- and AmpC-producing *E. coli* were isolated from faeces and farm dairy effluent in spring and summer, during months with varying levels of antimicrobial use, but no ESBL- or AmpC-producing *E. coli* were isolated from bulk tank milk or soil from recently grazed paddocks. Hybrid assemblies using short- and long-read sequence data from a subset of ESBL- and AmpC-producing *E. coli* enabled the assembly and annotation of nine plasmids from six *E. coli*, including one plasmid co-harbouring 12 antimicrobial resistance genes. ESBL-producing *E. coli* were infrequently identified from faeces and farm dairy effluent on the two NZ dairy farms, suggesting they are present at a low prevalence on these farms. Plasmids harbouring several antimicrobial resistance genes were identified, and bacteria carrying such plasmids are a concern for both animal and public health. AMR is a burden for human, animal and environmental health and requires a holistic “One Health” approach to address.

## Introduction

Antimicrobial resistance (AMR) is a complex “One Health” issue which affects human, animal, and environmental health (Queenan et al., 2016). Antimicrobial exposure, particularly the misuse and overuse of antimicrobials, in both human and animal health has been suggested as the main driver of AMR (Laxminarayan et al., 2013; Holmes et al., 2016). However, other factors such as heavy metal (Knapp et al., 2011; Chen et al., 2019; Thomas IV et al., 2020) and biocide use (Romero et al., 2017), and in the dairy farm environment, management practices such as waste milk disposal and feed type, may also influence the development and dissemination of AMR (Collis et al., 2019).

Extended-spectrum β-lactamase- (ESBL) and AmpC-producing *Escherichia coli* are of concern to human health. ESBL and AmpCs are β-lactamase enzymes which confer resistance to first- and third-generation cephalosporins (3GCs), penicillins, and monobactams. The AmpC β-lactamase enzymes also confer resistance to the second-generation cephalosporins and cephamycins (Rubin and Pitout, 2014). ESBL genes are often encoded on plasmids which can carry multiple antimicrobial resistance genes (ARGs), resulting in a multi-drug resistant phenotype (Ho et al., 2013; McGann et al., 2016). Therefore ESBL-producing *Enterobacteriaceae* have been classified as critical on the World Health Organisation’s “Priority Pathogens List” (Tacconelli et al., 2018). The most common ESBL variants include the SHV and TEM (excluding the parent type) enzymes and the CTX-M variants (Pitout, 2012). The predominant ESBL variants differ geographically and can exhibit temporal shifts (Livermore et al., 2006; Canton et al., 2008; Bevan et al., 2017), however, CTX-M-14 and CTX-M-15 are the predominant genotypes in most geographic regions (Bevan et al., 2017). In the livestock and animal sectors, the CTX-M-1, CTX-M-14, and CTX-M-15 ESBL types have frequently been detected (Ewers et al., 2012, 2021; Day et al., 2016; Afema et al., 2018; Cormier et al., 2019). In *E. coli*, an AmpC phenotype can arise from mutations in the promoter region of the chromosomal *ampC* gene resulting in AmpC hyperproducers (Caroff et al., 1999, 2000; Siu et al., 2003), or from plasmid-mediated AmpC β-lactamase (pAmpC) genes (Pérez-Pérez and Hanson, 2002). Plasmid-mediated resistance is of particular concern as this phenotype can be shared by horizontal gene transfer (HGT) between related bacteria. In addition, amino acid (AA) substitutions in the R2, H-9 and H-10 regions of chromosomal AmpC have been proposed to result in conformational changes within the enzyme, resulting in extended-spectrum AmpC β-lactamases (ESAC) which confer resistance against fourth-generation cephalosporins (4GCs) such as cefepime (Haenni et al., 2014a; Santiago et al., 2018). AA deletions in the H-10 helix have also been reported for plasmid-borne AmpC β-lactamases (CMY-33), which may alter the enzyme’s substrate spectrum (Pires et al., 2015).

Globally, ESBL- and AmpC-producing *Enterobacteriaceae* have been detected in various agricultural environments including dairy (Gonggrijp et al., 2016; Santman-Berends et al., 2017; Alzayn et al., 2020; Massé et al., 2021), poultry (Daehre et al., 2018; von Tippelskirch et al., 2018; Gazal et al., 2021), swine (von Salviati et al., 2015; Dohmen et al., 2017) and aquaculture (Hamza et al., 2020). The development and transmission of AMR in agricultural environments is complex. Risk factors for ESBL- and AmpC-producing *Enterobacteriaceae* on dairy farms includes 3GC and 4GC use, increased antimicrobial use in calves (Gonggrijp et al., 2016) and amoxicillin use for AmpC-producing *E. coli* (Alzayn et al., 2020). ESBL- and AmpC-producing *E. coli* have also been detected in organic dairy herds with low antimicrobial usage (AMU) (Santman-Berends et al., 2017). A study using mixed effects logistic regression found that AMU only partially explained ESBL/AmpC positive samples on dairy farms in the Netherlands (Hordijk et al., 2019). These findings suggest that multiple factors are involved in the development and transmission of AMR in dairy farm environments.

Few studies have investigated the prevalence of ESBL and AmpC-producing *E. coli* in pasture-based dairy farm environments such as those found in New Zealand (NZ) where there is relatively low use of antimicrobials in food-producing animals (Hillerton et al., 2017, 2021). One regional cross-sectional study of dairy farms in NZ found a low prevalence of ESBL-producing *E. coli* (1 of 116; 0.9%) in pooled faecal samples (Burgess et al., 2021a). Similarly, *E. coli* with pAmpC genes were absent but 7.9% (9 of 114) of faecal samples were positive for AmpC hyperproducers with mutations in the promoter region of the *ampC* gene (Burgess et al., 2021a). A nationwide cross-sectional study (*n* = 26 dairy farms) in NZ did not detect any ESBL-producing *E. coli* (Burgess et al., 2021b) while AmpC-hyperproducing *E. coli* were isolated from 14% (11 of 78) of pooled faecal enrichments originating from seven farms (Burgess et al., 2021b). Additionally, an NZ study between 2009 and 2010 found no ESBL-producing *E. coli* (0 of 300) from young calf carcasses (4–10 days old) (Heffernan et al., 2011). Hence, the aims of this study were to utilise culture-based methods to investigate the prevalence of ESBL- and AmpC-producing *E. coli* from two NZ dairy farm environments over a 15-month period, taking into consideration seasonal variation and farm management practices, and to understand the genomic epidemiology and ARG repertoire of a subset of 3GC resistant *E. coli* isolated in this study.

## Materials and methods

### Study population and sample collection

The Massey University research farms No. 1 Dairy Farm (referred to hereafter as Dairy 1) and No. 4 Dairy Farm (referred to hereafter as Dairy 4) were recruited for inclusion in this study. The two dairy farms are located in Palmerston North, New Zealand, are <5 km apart and both operate a closed dairy farm system (animals are not introduced into the herd). The two farms are pasture-based, with the use of supplementary feed such as silage (pickled pasture) and baleage (partially dried pasture) when required. Both dairy farms have a spring calving system, use selective dry cow therapy (DCT) and teat sealants are applied to the whole herd. A comparison between farm parameters and management practices is detailed in Supplementary Table 1.

Farm environmental samples were collected concurrently each month from October 2018 to December 2019, spanning a 15-month period and both farms were sampled on the same day. On each sampling occasion, composite soil cores and composite cow faeces from cow pats in a recently grazed paddock, farm dairy effluent (FDE) and bulk tank milk (referred to hereafter as milk) were collected (Supplementary Table 2). Previous studies looking at the prevalence of antimicrobial resistant *E. coli* in soil from dairy farms have also utilised a sampling strategy which focuses on critical source areas where a large number of dairy cows are concentrated or areas recently impacted by dairy cows, such as housing areas, gateways, recently grazed paddocks and manure fertilised soil (Liu et al., 2019; Gelalcha et al., 2022). The FDE management strategy on Dairy 1 changed during the study period; initially effluent was filtered before being administered to paddocks, but later effluent was disposed of via the municipal waste-water system and as a result, two FDE sample collection points were used (Supplementary Table 2). Samples were transported to the Hopkirk Research Institute (Massey University, Palmerston North, New Zealand) and stored at 4°C until processing (within 8 h of collection). Farm management practice metadata were collected during sampling visits. AMU was reported as individual animal antimicrobial treatments recorded on farm. The total amount administered for each treatment (mg) was calculated according to the concentration of the product (mg/ml), number of doses and volume (ml). AMU was reported as mg/population correction unit (PCU) calculated using the total active ingredient weight (mg)/herd size/average cow size in NZ (453 kg).

### Sample processing

The environmental samples were enriched in buffered peptone water (BD Difco,™ Fort Richard Laboratories, Auckland, New Zealand) at 35°C for 18 h (Supplementary Table 2). After incubation, enrichments were mixed by vortex and 1 ml was mixed with glycerol (30% [v/v]) and stored at −80°C.

### Microbiological methods

Frozen enrichments (Dairy 1; *n* = 101, Dairy 4; *n* = 103) were plated on (i) MacConkey (MC) agar plates (Fort Richard Laboratories, Auckland, New Zealand) as a positive control to ensure growth of *E. coli*, (ii) in-house prepared MC agar (BD Difco™) plates supplemented with 1 μg/ml cefotaxime sodium (Sigma-Aldrich, St. Louis, MO, United States), (iii) MC agar (BD Difco™) plates supplemented with 1 μg/ml ceftazidime (Sigma-Aldrich) and (iv) CHROMagar ESBL™ (CHROMagar, Paris, France, Fort Richard Laboratories), as previously described (Burgess et al., 2021a), except Columbia Sheep Blood agar (5% blood) (Fort Richard Laboratories) was used for the purification of isolates.

Presumptive pure *E. coli* isolates were stored in in-house prepared brain heart infusion broth (Oxoid, Hampshire, United Kingdom) containing glycerol (30% [v/v]) at −80°C. Isolates were identified by matrix-assisted laser desorption/ionization-time of flight (MALDI-TOF) mass spectrometry (MS) (Bruker Daltonics, Billerica, CA, United States) using the previously described “on slide formic acid extraction” method (Lévesque et al., 2015). Confirmed *E. coli* strains isolated from agars (ii – iv) outlined above were subjected to antimicrobial susceptibility testing (ASTs) against six antimicrobials (Supplementary Table 3) using the Kirby-Bauer disc diffusion tests and CLSI guidelines (CLSI, 2019). An AmpC and ESBL positive phenotype was confirmed using either a three-disc (D69C AmpC disc test, Mast Group Ltd., Liverpool, United Kingdom) or double-disc comparison assay (D62C cefotaxime and D64C ceftazidime ESBL disc tests, Mast Group Ltd., Liverpool, United Kingdom), respectively. Isolates which were AmpC positive and had a zone size which could not be differentiated as either positive or negative (≥2–≤5 mm) for either ESBL double-disc comparison assay were further tested using a double-disc assay containing cefepime (D63C cefepime ESBL disc test, Mast Group Ltd., Liverpool, United Kingdom). The AmpC-producing *E. coli* isolate NZRM4402 and the ESBL-producing *Klebsiella pneumoniae* isolate NZRM3681 were used as positive controls in the AmpC and ESBL confirmatory disc assays, respectively, and the susceptible *E. coli* isolate NZRM916 was used as a negative control.

### Molecular characterisation

Crude DNA was extracted from pure isolates by adding three or four colonies to 400 μl sterile molecular biology-grade water and heating at 100°C for 10 min in a heating block and storing at −20°C. The supernatant was used for subsequent PCR reactions and the PCR conditions are detailed in Supplementary Table 4. The *E. coli* phylogroup was determined using the Clermont quadruplex PCR Typing method (Clermont et al., 2000, 2013) with KAPA Hifi HotStart ReadyMix (KAPA BioSystems, Wilmington, United States). *E. coli* with an AmpC phenotype were tested for pAmpC gene families using a multiplex PCR (Pérez-Pérez and Hanson, 2002) and a PCR targeting the *bla*_CMY_ gene family (Dierikx et al., 2012) was used on boiled DNA preparations from isolates positive for the CITM primer set, indicative of CMY-positive *E. coli*. *E. coli* in which no pAmpC genes were identified were further analysed to identify mutations in the promoter region of the *ampC* gene (Caroff et al., 1999). PCR products were purified using the QIAquick PCR purification kit (QIAGEN, Hilden, Germany) and the sequencing reaction primers and conditions are detailed in Supplementary Table 4. Capillary separation of sequencing reactions was undertaken by the Massey Genome Service using an ABI3730 DNA analyser (Massey University, Palmerston North, New Zealand). DNA sequence chromatograms were trimmed using Geneious Prime v2019.1.1 (Kearse et al., 2012; Biomatters Geneious Prime, 2022) and the complete PCR products were confirmed against the NCBI database using BLASTN (Altschul et al., 1990). The ESBL *bla*_CTX–M_ group was confirmed using the CTX-M-1-group PCR (Gonggrijp et al., 2016) for the ESBL-producing *E. coli* which did not undergo whole genome sequencing (WGS). PCR reactions were performed using HOT FIREPol^®^ Blend Master Mix (Solis BioDyne, Tartu, Estonia) unless stated otherwise.

### DNA extraction, library preparation, and whole genome sequencing

A subset of ESBL- and AmpC-producing *E. coli* were selected for WGS according to phenotype, resistance profile, *E. coli* phylogroup and metadata (including farm, source, and collection date). For samples where multiple isolates were identified, only one isolate per molecular and resistance profile combination was included. Bacterial isolates from glycerol broths stored at −80°C were inoculated on Columbia Sheep Blood agar (5% blood) and incubated for 18 h at 35°C. An individual colony was subsequently sub-cultured onto a fresh Columbia Sheep Blood agar plate (5% blood) and incubated at 35°C for 18 h. An individual colony was inoculated in 4 ml Luria-Bertani Miller broth (Fort Richard Laboratories) and incubated at 35°C for 15 h at 200 rpm. Genomic DNA (for both Illumina and MinION sequencing methods) was extracted using the Wizard^®^ Genomic DNA Purification Kit (Promega, Madison, WI, United States), according to the manufacturer’s instructions for Gram-negative bacteria. Several modifications were included to optimise the protocol for *E. coli*. Briefly, 2 ml overnight culture was centrifuged at 13,000 × *g* for 2 min to pellet the cells which were treated with RNAse (100 mg/ml) at 37°C for 1 h. After the Protein Precipitation Solution was added to the cell lysate and incubated on ice for 5 min, the sample was centrifuged at 13,000 × *g* for 3 min, the supernatant transferred to a clean tube and centrifuged again at 13,000 × *g* to reduce any residual protein contamination. The DNA was re-hydrated overnight at 4°C in 100 μl 10 mM TrisHCl pH 8.5 (Geneaid Biotech Ltd., New Taipei City, Taiwan).

The DNA concentration was quantified using a Qubit 4.0 fluorometer (Thermo Fisher Scientific Inc., Waltham, MA, United States) and *A*_260/280_ and *A*_260/230_ ratios determined using the Nanodrop microvolume spectrophotometer (Nanodrop 2000c, Thermo Fisher Scientific Inc.). DNA integrity and size was visualised on a 0.8% [w/v] agarose gel using a high molecular weight Lambda DNA/*Hin*dIII ladder (Thermo Fisher Scientific Inc.). The libraries were prepared using the Nextera XT DNA library preparation kit (Illumina, San Diego, CA, United States) and sequencing performed using an Illumina MiSeq v3 with 2 × 300 bp paired-end reads (Massey Genome Service, Massey University, Palmerston North, New Zealand). The Nanopore MinION sequencing was performed at the Molecular Epidemiology and Public Health Laboratory (Massey University, Palmerston North, New Zealand) using a R9.4.1 flow cell (Oxford Nanopore Technologies, United Kingdom). The libraries were prepared using the Rapid Barcoding Sequencing kit (SQK-RBK004; Oxford Nanopore Technologies, United Kingdom) according to the manufacturer’s instructions (“Library Preparation”), with minor modifications for *E. coli*. Briefly, 600 ng DNA template was used as input and the DNA pellet was resuspended in 10 μl 10 mM TrisHCl/50 mM NaCl by incubating at 50°C for 10 min. The flow cell was primed and loaded according to the manufacturer’s instructions and run for 24–48 h (SQK-RBK004, “Priming and loading the SpotON flow cell,” Oxford Nanopore Technologies, United Kingdom).

### Bioinformatics and data analysis

The Illumina MiSeq sequencing reads were randomly subsampled down to a 100× genome sequencing depth using Rasusa v0.6.0 (Hall, 2022). The Illumina MiSeq sequencing reads were subsequently processed using the Nullarbor v2.0 (Seemann et al., (n.d.)) pipeline with default parameters. In summary, adapters were removed from raw reads using Trimmomatic v0.39 (Bolger et al., 2014), species identification by *k*-mer analysis performed using the Kraken v1.1.1 database (Wood and Salzberg, 2014), the genomes were assembled using SKESA v2.4.0 (Souvorov et al., 2018) and annotated with Prokka v1.14.6 (Seemann, n.d.a). The sequence type was determined using mlst v2.19.0 (Seemann, n.d.b) with information downloaded from PubMLST (Jolley and Maiden, 2010), the resistome profile identified with ABRicate v1.0.1 (Seemann, n.d.a) using the Resfinder 4.0 database (Zankari et al., 2012; Bortolaia et al., 2020), and the Centre for Genomic Epidemiology website (Center for Genomic Epidemiology, 2011) was used to detect the virulence genes and serotype in assembled genomes using the VirulenceFinder 2.0.3 database (v2020-05-29) (Joensen et al., 2015) and the SeroTypeFinder 2.0.1 database (v1.0.0), respectively. The presence/absence data from 37 virulence associated genes identified in the whole genome sequences (*n* = 12) was used to construct a hierarchical cluster tree using Jaccard distances and the tree was further annotated using the Interactive Tree of Life webserver (Letunic and Bork, 2016). The core single nucleotide polymorphism (SNP) variation was assessed using Snippy v4.4.3 (Seemann, 2016) with a ST131 ESBL-producing *E. coli* JJ1887 as the reference sequence (Genbank accession: CP014316). A maximum-likelihood tree was generated from the core SNP alignment using a general time-reversible model with the Randomised Axelerated Maximum Likelihood (RAxML) Next-Generation tool (Kozlov et al., 2019) and visualised in GrapeTree (Zhou et al., 2018).

For long-read data, the MinION fast5 sequencing read files were basecalled using Guppy v4.2.2. The reads were de-multiplexed using qcat v1.1.0 (Oxford Nanopore Technologies, 2019) and adapters removed with Porechop v0.2.4 (Wick, 2018b) using default settings. Filtlong v0.2.0 (Wick, 2018a) was used to trim the reads with a minimum length of 1 kb. Hybrid assemblies were generated using Unicycler v0.4.9b (Wick et al., 2017) with default settings. Plasmids from hybrid assemblies were re-constructed and typed using MOB-suite v1.4.9.1 (Robertson and Nash, 2018; Robertson et al., 2020) and annotated with Prokka v1.14.6 (Seemann, n.d.a) using a custom database consisting of the best-match “nearest neighbour” plasmids as identified by MOB-suite v1.4.9.1 (Genbank accessions: CP009566, CP015997, CP018107, CP016585, and KF362121). For pMLST, variants within IncI group plasmids from hybrid assemblies and plasmid incompatibility groups for short-read data only were identified (Carattoli et al., 2014). Plasmid core genome variation for selected isolates was assessed using Snippy v4.6.0 (Seemann, 2016) with pDF0049.2e_1 as the reference. Clusters of Orthologous Groups (COG) were identified using eggNOG-mapper v2 (Huerta-Cepas et al., 2017, 2019; Cantalapiedra et al., 2021). The plasmid *oriC* region was identified using DoriC v10.0 (Luo and Gao, 2019). Statistical tests were performed in Minitab^®^ 19.1.1 (Minitab, 2019) using a one-way analysis of variance (ANOVA) with a 95% confidence interval for sample prevalence comparisons.

## Results

Farm samples were obtained from Massey University research farms Dairy 1 and Dairy 4 over a 15-month period from October 2018 to December 2019, consisting of composite faeces (*n* = 60), milk (*n* = 13), soil (*n* = 15), and FDE (*n* = 13 Dairy 1; *n* = 15 Dairy 4) from each farm. From the selective agar plates, 52 putative 3GC resistant *E. coli* (*n* = 24 Dairy 1; *n* = 28 Dairy 4) were isolated (Supplementary Table 5).

### Antimicrobial susceptibility profiles

**FIGURE 1:**
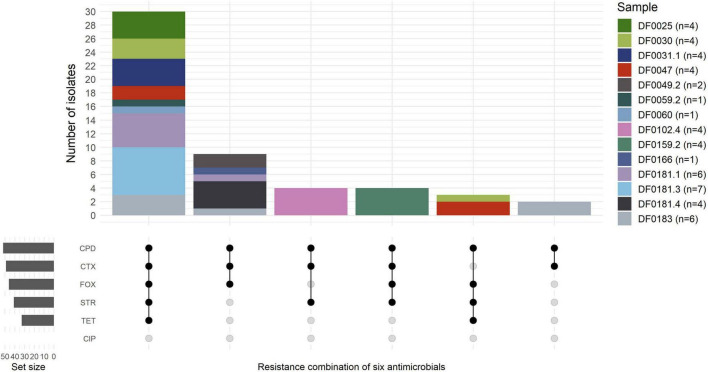
Resistance profiles of *E. coli* (*n* = 52) isolated across 14 farm samples. The number of isolates per sample is indicated in the brackets. Isolates with intermediate resistance to cefotaxime (*n* = 15) were grouped as resistant in the Upset plot. CPD, cefpodoxime (10 μg); FOX, cefoxitin (30 μg); STR, streptomycin (10 μg); CTX, cefotaxime (30 μg); TET, tetracycline (30 μg); CIP, ciprofloxacin (5 μg).

The susceptibility of *E. coli* isolated in this study (*n* = 52) to six clinically relevant antimicrobials, including three β-lactams, was examined (Figure 1 and Supplementary Table 6). All of the *E. coli* (*n* = 52) were resistant to cefpodoxime, 46 of 52 (89%) to cefoxitin and 34 of 52 (65%) were resistant or intermediate (15 of 52; 29%) to cefotaxime. Numerous isolates were resistant to streptomycin (41 of 52; 79%) and tetracycline (33 of 52, 64%) although all isolates (*n* = 52) were susceptible to ciprofloxacin, which is a critically important antimicrobial in human medicine (World Health Organization, 2011). The susceptibility of 20 *E. coli* to the 4GC cefepime was assessed (Supplementary Table 6) representing at least one *E. coli* from every sample and all phylotype and resistance profile combinations. Seven isolates from four samples were cefepime resistant and the remaining 13 *E. coli* were either susceptible or intermediate (Supplementary Table 6). According to phenotypic testing, 33 of 52 *E. coli* (64%) were multi-drug resistant, but phenotypic testing was only performed using antibiotics representing four classes (β-lactams, tetracyclines, aminoglycosides, and fluoroquinolones).

At the isolate level, all putative antimicrobial resistant *E. coli* (*n* = 52) were tested for AmpC and ESBL production. In total, 46 of 52 (88.5%) were AmpC-producing *E. coli* and, 2 of 52 (3.8%) isolates from the same FDE sample were confirmed as ESBL producers (DF0183c and DF0183g), and one (1.9%) isolate (DF0059.2e) was both ESBL and AmpC positive. Four isolates (7.7%) from the same sample (DF0102.4e-h) were both ESBL and AmpC negative according to the phenotypic tests.

### Prevalence of extended-spectrum β-lactamase- and AmpC-producing *E. coli* from farm environmental samples

Farm samples were investigated for ESBL- and AmpC-producing *E. coli* (Table 1). At the sample level, one of 60 (1.7%) pooled faecal and one of 15 (6.7%) FDE samples from Dairy 4 were positive for ESBL-producing *E. coli*, with one and two isolates cultured from these samples, respectively. No ESBL-producing *E. coli* were isolated from pooled faeces and FDE from Dairy 1 nor from soil from the most recently grazed paddocks or milk samples from either farm. AmpC-producing *E. coli* were isolated from faeces (2 of 60, 3.3%; 5 of 60, 8.3%) and FDE (5 of 13, 38.5%; 1 of 15, 6.7%) on Dairy 1 and Dairy 4, respectively and none were isolated from soil (0 of 30) or milk (0 of 26) from either farm. The sample level prevalence of AmpC-producing *E. coli* isolated from Dairy 1 and Dairy 4 (*p* = 0.526) and between sample types (faeces or FDE; *p* = 0.408) was not statistically significant. ESBL-producing *E. coli* were isolated at a low prevalence on Dairy 4 but were not isolated from Dairy 1 during this study period. Dairy 4 had a higher prevalence of AmpC-producing *E. coli* from faeces (8.3%), whereas Dairy 1 had a higher number isolated from FDE (38.5%).

**TABLE 1:** Number of positive ESBL- and AmpC-producing *Escherichia coli* samples and isolates.

Farm	Sample type^a^	ESBL-producing *E. coli*^b^		AmpC-producing *E. coli*	
		No. samples (%)	No. isolates	No. samples (%)	No. isolates
Dairy 1	FDE	0/13	0	5/13 (38.5%)	14
	Faeces	0/60	0	2/60 (3.3%)	6
	Milk	0/13	0	0/13	0
	Soil	0/15	0	0/15	0
Dairy 4	FDE	1/15 (6.7%)	2	1/15 (6.7%)	4
	Faeces	1/60 (1.7%)	1	5/60 (8.3%)	22
	Milk	0/13	0	0/13	0
	Soil	0/15	0	0/15	0

^a^FDE, farm dairy effluent. ^b^One isolate was both ESBL and AmpC positive (DF0059.2e) and has been included in both columns.

### Estimated antimicrobial use on dairy farms between October 2018 and December 2019

According to antimicrobial treatment data, between October 2018 and December 2019 the estimated AMU on Dairy 1 and Dairy 4 was 17.09 mg/PCU and 5.36 mg/PCU, respectively (Supplementary Tables 7,8). Months with higher AMU are consistent with spring calving in NZ. The New Zealand Veterinary Association (NZVA) has classified antimicrobials as green, yellow or red tier according to the World Health Organisation classes (Anonymous, 2018). Of the total AMU used on Dairy 1 and Dairy 4, the majority of antimicrobials were green tier (91.1 and 30.5%), followed by yellow tier (6.2 and 67.9%) while red tier antimicrobials were infrequently used (2.75 and 1.6%), respectively.

As shown in Figure 4, AmpC-producing *E. coli* were isolated in spring and summer and after months of both high and low AMU. On Dairy 1, the highest AMU occurred in October 2018 (12.3%) and August to November 2019 (8.7–18.6%); whereas on Dairy 4 the highest AMU predominantly occurred in September 2019 (56.7%) (Figure 4 and Supplementary Tables 7–9).

### Molecular characterisation of extended-spectrum β-lactamase- and AmpC-producing *E. coli*

At the isolate level, the predominant *E. coli* phylogroups were B1 (18 of 52, 35%) and C (17 of 52, 33%), followed by E (6 of 52, 12%), D (5 of 52, 10%), A (4 of 52, 8%), and F (2 of 52, 4%). The *bla*_CTX–M–15_ gene was identified in the ESBL-producing *E. coli* (DF0183c and DF0183g). Isolate DF0059.2e was confirmed as both AmpC and ESBL positive using phenotypic and genotypic testing and co-harboured the *bla*_CMY–2_ and *bla*_CTX–M–1_ genes. The plasmid-mediated gene *bla*_CMY–2_ was detected in 28 of 46 isolates (61%). Plasmid-mediated AmpC strains were predominantly isolated from Dairy 4 (27 of 28, 96%). For the remaining 18 AmpC-producing *E. coli*, mutations were identified in the promoter region of the *ampC* gene. All AmpC hyperproducers (*n* = 18) were isolated from Dairy 1. The 18 isolates were all phylogroup C and had mutations in the promoter region of the *ampC* gene at positions -42 (C → T), -18 (G → A), -1 (C → T) and +58 (C → T), excluding DF0025c in which position −42 could not be determined [positions relative to the *E. coli* K12 transcriptional start base (+1) (Olsson et al., 1982)]. No pAmpC or ESBL genes were detected in 3GC resistant four *E. coli* which were AmpC and ESBL negative in the phenotypic testing (DF0102.4e-h). Therefore, the mechanism for resistance to 3GCs was unassigned for these isolates (designated as ‘unknown’ in subsequent figures).

To determine whether the cefepime resistant phenotype of the five isolates which were not ESBLs was a result of extended-spectrum AmpC beta-lactamase production, the deduced AA sequence of AmpC was compared between isolates DF0102.4g (chromosomal AmpC), DF0159.2g (plasmid-mediated AmpC), previously identified ESAC producers and a negative control. AA substitutions were identified in the omega loop at positions 191 (K191Q) for both isolates and at positions 201 (N201T) and 209 (S209P) for DF0102.4g and DF0159.2g, respectively; DF0159.2g also had an AA substitution in the R2 loop region, at position 300 (I300V) (Table 2). Neither isolate had any substitutions in the H-9 or H-10 helices.

**TABLE 2:** Amino acid substitution profiles of suspected extended-spectrum AmpC β-lactamase producing *E. coli* from this study.

Strain^‡^	4*	8	105	130	140*	191	201*	209	236	248	251	254	255	256	257	260*	261	300	316	370^†^
ATCC 25922	T	T	T	A	E	K	N	S	A	R	Q	L	K	P	L	N	E	I	P	W
DF0159.2g	T	A	T	T	E	Q	N	P	T	C	R	M	N	H	R	N	D	V	A	W
DF0102.4g	M	A	A	A	D	Q	T	S	A	R	Q	L	K	P	L	T	E	I	A	C
F8	T	A	A	A	E	Q	N	P	A	R	Q	L	K	P	L	N	E	V	A	ND
F21	T	A	T	T	E	Q	N	P	T	C	R	M	N	H	R	N	D	V	A	ND
N18	T	A	A	A	E	Q	N	P	A	R	Q	L	K	P	L	N	E	V	A	ND

^‡^E. coli ATCC25922 negative control (accession number: NZ_CP009072). F8, F21, N18 suspected extended-spectrum AmpC β-lactamase producers isolated from bovine faeces and milk in Brazil (Santiago et al., 2018). *New AA substitution described. Positions 191, 201, and 209 in omega loop. Position 300 is within the R2 loop. ^†^No data was available for this position.

### Population structure and comparative genomics

A subset of 3GC resistant *E. coli* were analysed using WGS to understand the genomic epidemiology and transmission dynamics of these bacteria on farm (Table 3). *E. coli* isolates with a plasmid-mediated AmpC-producing phenotype (*n* = 4), ESBL-producing (*n* = 1), ESBL/AmpC-producing (*n* = 1) and an unassigned mechanism for resistance to 3GCs (*n* = 1) were selected for Oxford Nanopore Technologies (ONT) MinION long-read sequencing, with a focus on generating complete genomes and examining plasmids in detail. The population structure of the AmpC- and ESBL-producing *E. coli* which were sequenced in this project (*n* = 12) was diverse and the isolates belonged to eight sequence types including ST56 (3 of 12, 25%), ST57 (2 of 12, 17%), ST88 (2 of 12, 17%) and singletons for ST442, ST973, ST2541, ST4553, and ST5135 (1 of 12, 8%) (Table 3). All isolates had a genome size ranging from 4,839,855 to 5,431,661 bp and a GC content between 51.7 and 52.0%.

**TABLE 3:** Genome composition of *E. coli* (*n* = 12) sequenced in this study.

Isolate^a^	Farm	Source^b^	Collection date	Phylo-group	Sequence type	Serotype^c^	Phenotype/ Genotype^d^	Genome size (bp)	Contigs^e^	GC (%)	Plasmids
DF0031.1c	1	Faeces	12/2018	C	88	O8/O32:H19	AmpC	5,180,929	91	51.9	IncFIB, IncFII, IncQ1
DF0047c	1	FDE	01/2019	C	88	O8/O32:H19	AmpC	5,157,783	90	51.9	IncFIB, IncFII, IncQ1
DF0049.2e*^a^*	1	Faeces	01/2019	E	57	O27:H18	pAmpC	5,047,883	86 (2*)	51.8	IncI1
DF0059.2e*^a^*	4	Faeces	02/2019	D	5135	ONT:H26	pAmpC/ESBL	5,437,661	99 (4*)	51.7	IncI1, IncY, IncFIA/IncQ1
DF0102.4g*^a^*	1	Faeces	05/2019	D	973	ONT:H15	Unknown	5,041,356	81 (4)	51.7	IncI1, IncI1/IncFII
DF0159.2g*^a^*	4	Faeces	10/2019	A	2541	ONT:H7	pAmpC	4,869,780	80 (5)	51.9	IncI1
DF0181.1c*^a^*	4	Faeces	12/2019	B1	56	O8/O40:H21	pAmpC	5,199,529	192 (3)	51.9	IncI1
DF0181.3c*^a^*	4	Faeces	12/2019	B1	56	O8/O40:H21	pAmpC	5,202,415	194	52.0	IncI1
DF0181.4c	4	Faeces	12/2019	E	57	O124/O164: H25	pAmpC	4,961,348	83	51.9	IncI1
DF0183e*^a^*	4	FDE	12/2019	B1	56	O8/O40:H21	pAmpC	5,201,499	194 (2*)	51.8	IncI1
DF0183g	4	FDE	12/2019	F	4553	O83:H42	ESBL	5,001,896	70 (1*)	51.8	None
DF0183i	4	FDE	12/2019	B1	442	O146:H21	pAmpC	4,839,955	69	51.8	IncI1/IncFIB

^a^Isolates sequenced using both Illumina MiSeq and Oxford Nanopore Technologies. ^b^FDE, farm dairy effluent. ^c^ONT, O non-typeable. According to SerotypeFinder, the O serogroup was non-typeable for three strains. ^d^Resulting phenotype/genotype, pAmpC, Plasmid-mediated AmpC β-lactamase; AmpC, E. coli with mutations in the promoter region of the ampC gene. ^e^Hybrid assembly contig numbers in brackets. Asterisk indicates a closed genome.

**FIGURE 2:**
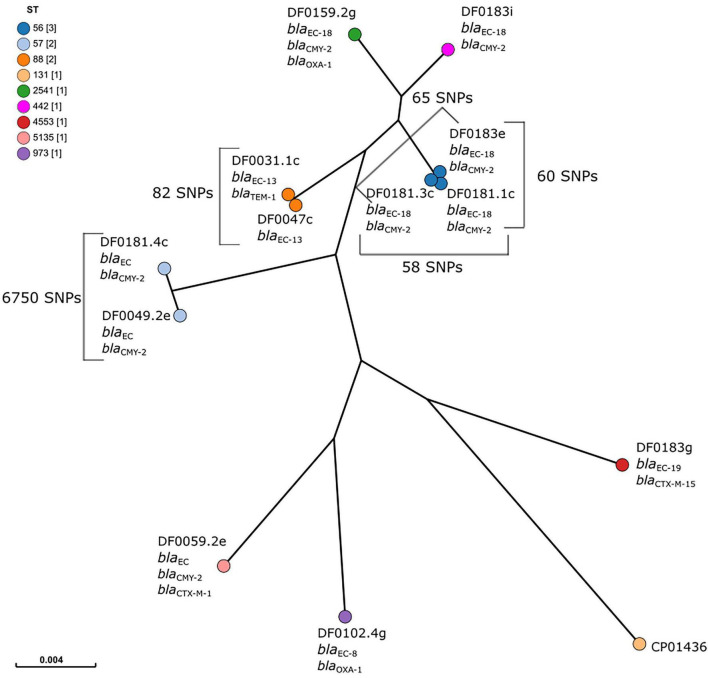
Maximum-likelihood tree of core genome single nucleotide polymorphism (SNP) analysis of ESBL and AmpC-producing *E. coli* (*n* = 12). *E. coli* CP014316 (ST131, *bla*_*CTX–M–*15_ positive) was used as the reference and nodes are coloured by sequence type, as indicated in the figure legend. The scale bar indicates the proportion of the core genome alignment over which core SNPs have been calculated. The number of SNPs between isolates in the same cluster is indicated on the figure.

Core genome SNP analysis separated the strains into eight clusters, each representing a different ST (Figure 2). The separation of clusters according to ST was reflected in the large number of SNPs identified in the core genome of these strains (102,841 SNPs), accounting for approximately 2.0% of the *E. coli* genome. However, within two clusters genetically similar isolates representing two STs were identified. Three *bla*_CMY–2_-positive *E. coli* were isolated in December 2019 on Dairy 4 from two faeces and one FDE sample, all belonged to ST56 and were genetically similar (58 – 65 SNPs; isolates DF0181.1c, DF0181.3c, and DF0183e). Similarly, two AmpC hyperproducers, were isolated in December 2018 and January 2019 from Dairy 1 and differed by 82 SNPs (DF0031.1c and DF0047c). In each case, the *E. coli* were isolated from both faeces (ST56 *n* = 2; ST88 *n* = 1) and FDE (ST56 *n* = 1; ST88 *n* = 1).

### Antimicrobial resistance genes

Analysis of sequence data of 12 *E. coli* confirmed the presence of the β-lactam resistance genes *bla*_CMY–2_ (8 of 12, 66.7%), *bla*_CTX–M–1_ and *bla*_CTX–M–15_ (1 of 12 each, 8.3%) and *bla*_OXA–1_ (2 of 12, 16.7%) (Table 4). The genotypic results were in agreement with the confirmed ESBL and AmpC phenotypes. All isolates carried the *ampC* gene (a synonym for the *bla*_EC_ gene (Mammeri et al., 2006) and gene-specific variation observed was broadly associated with the different *E. coli* phylogroups.

**TABLE 4:** Resistance profiles of ESBL- and AmpC-producing *E. coli* (*n* = 12).

Isolate	AST^a^ phenotype	β-lactam resistance genes^b^	Other ARGs^c^
DF0031.1c	CPD, FOX, TET, STR	*bla*EC-13, *bla*TEM-1	*aph(3*′′*)-Ib*, *aph(3*′*)-Ia*, *aph(6)-Id*, *dfrA5*, *sul1*, *sul2*, *tet(A)*
DF0047c	CPD, FOX, TET, STR	*bla*EC-13	*aph(3*′′*)-Ib*, *aph(3*′*)-Ia*, *aph(6)-Id*, *sul2*, *tet(A)*
DF0049.2e	CTX, CPD, FOX	*bla*EC, *bla*CMY-2	None
DF0059.2e	CTX, CPD, FOX, TET, STR	*bla*EC, *bla*CMY-2, *bla*CTX-M-1	*aac(3)-IId*, *aadA5*, *aph(3*′′*)-Ib*, *aph(3*′*)-Ia*, *aph(6)-Id*, *catA1*, *dfrA17*, *mph(A)*, *sul1*, *sul2*, *tet(B)*
DF0102.4g	CTX, CPD, STR	*bla*EC-8, *bla*OXA-1	*aadA1*, *sul1*
DF0159.2g	CTX, CPD, FOX, STR	*bla*EC-18, *bla*CMY-2, *bla*OXA-1	*aadA1*, *sul1*
DF0181.1c	CTX, CPD, FOX, TET, STR	*bla*EC-18, *bla*CMY-2	*aph(3*′′*)-Ib*, *aph(6)-Id*, *sul2*, *tet(B)*
DF0181.3c	CTX, CPD, FOX, TET, STR	*bla*EC-18, *bla*CMY-2	*aph(3*′′*)-Ib*, *aph(6)-Id*, *sul2*, *tet(B)*
DF0181.4c	CTX, CPD, FOX	*bla*EC, *bla*CMY-2	None
DF0183e	CTX, CPD, FOX, TET, STR	*bla*EC-18, *bla*CMY-2	*aph(3*′′*)-Ib*, *aph(6)-Id*, *sul2*, *tet(B)*
DF0183g	CTX, CPD	*bla*EC-19, *bla*CTX-M-15	None
DF0183i	CTX, CPD, FOX	*bla*EC-18, *bla*CMY-2	None

^a^Antimicrobial susceptibility testing; CPD, cefpodoxime; FOX, cefoxitin; STR, streptomycin; CTX, cefotaxime; TET, tetracycline. ^b^bla_EC_ is a synonym for the ampC gene. ^c^Antimicrobial resistance genes defined in Supplementary Table 10.

Other ARGs including *mph(A)*, *catA1*, *dfrA5*, *dfrA17*, *sul1*, and *sul2* were identified during analysis of the assembled genomes but representative phenotypes were not examined. Initial PCR methods failed to identify any ESBL and AmpC genetic determinants associated with four DF0102.4e-h isolates resistant to 3GC and 4GCs (cefotaxime, cefpodoxime, and cefepime). Further analysis of DF0102.4g using WGS also failed to determine the genetic basis for the AMR phenotype. However, this isolate carried the *bla*_OXA–1_ gene which encodes a narrow-spectrum Class D β-lactamase and had several AA substitutions in the chromosomally encoded AmpC, although no substitutions were identified in the R2 loop of H-9 or H-10 helices. According to the phenotypic confirmation tests, this isolate was AmpC negative and this phenotype was confirmed by *in silico* analysis of the promoter region of the *ampC* gene which failed to identify any mutations in the promoter elements, although mutations within the *ampC* gene (Caroff et al., 1999) were identified at positions +70 (C → T) and +81 (A → G).

### Virulence factors and *E. coli* pathotypes

**FIGURE 3:**
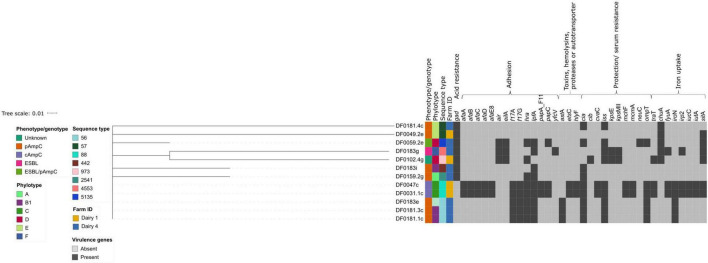
Hierarchical cluster tree constructed using Jaccard distances for the presence or absence data from 37 virulence genes identified using Virulence Finder. The tree was edited using the Interactive Tree of Life webserver. Isolate metadata is included for ESBL or AmpC phenotype, farm and sequence type, as indicated by the colour keys. The virulence genes are grouped and annotated by general function.

The *E. coli* sequenced in this study carried a range of virulence factors, which were mainly involved in adhesion, protection/serum resistance, iron uptake and toxins, hemolysins, proteases or autotransporters (Figure 3). *E. coli* strains of the same ST clustered together, based on the presence/absence of virulence factors. The number of virulence factors each *E. coli* harboured varied, with strains DF0049.2e and DF0183i carrying the fewest virulence factors (3 of 139) and DF0031.1c and DF0047c carrying the most (28 of 139).

**FIGURE 4:**
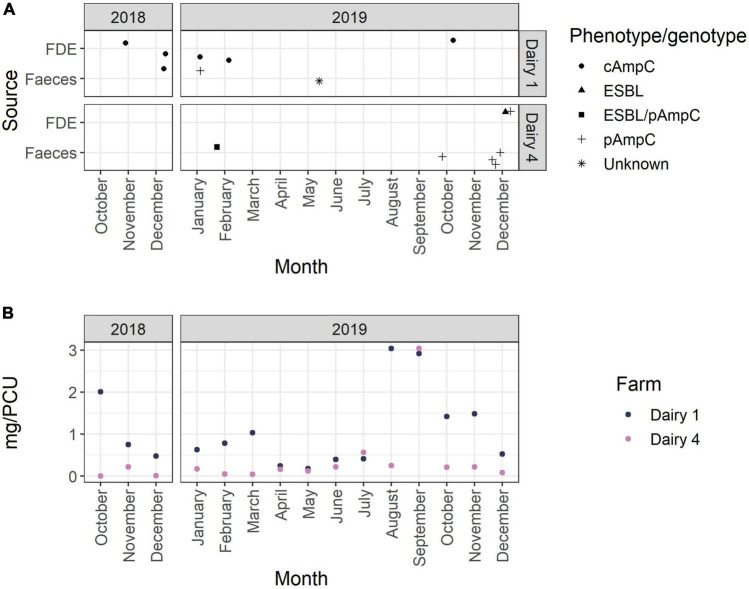
**(A)** Collection dates of samples positive for ESBL- and/or AmpC-producing *E. coli* over the 15-month study period. The phenotype is represented by shape as indicated in the figure legend. **(B)** Antimicrobial use per month (mg active ingredient per population correction unit) on Dairy 1 and Dairy 4 during the 15-month study period.

Five *E. coli* strains were putatively classified as avian pathogenic *E. coli* (APEC), a subgroup of extraintestinal pathogenic *E. coli*, due to the presence of the following virulence factors: salmochelin siderophore receptor (*iroN*), outer membrane protease (*ompT*) and increased serum survival (*iss*) (in DF0181.1c, DF0181.3c, and DF0183e) as well as ferric aerobactin receptor (*iutA*) and avian hemolysin (*hlyF*) which were also detected in strains DF0031.1c and DF0047c. These virulence factors have been associated with APEC and are often encoded on plasmids (Johnson et al., 2008). No *E. coli* harboured the Shiga toxin genes (*stx1* or *stx2*) or the locus of enterocyte effacement pathogenicity island, indicative of Shiga-toxin producing *E. coli* (STEC) or enteropathogenic *E. coli* (EPEC), respectively (Robins-Browne et al., 2016). Three strains carried the *air* gene for the enteroaggregative immunoglobulin repeat protein Air which is commonly found in enteroaggregative *E. coli* (EAEC), however, other virulence genes such as *EAST-1* and *pic* associated with this pathotype were not detected (Johnson et al., 2008). Five strains harboured enterotoxigenic *E. coli* (ETEC) associated fimbriae F17 (*f17A* and *f17G*) encoding genes, although no other ETEC-related toxin or fimbriae genes were detected in these strains.

### Plasmid characteristics

A large proportion of the AmpC- (8 of 10, 80%) and ESBL-encoding (1 of 2, 50%) genes identified from *E. coli* isolated within this study are plasmid-associated. Four isolates sequenced using MinION long-read methods carried one IncI1 plasmid harbouring the *bla*_CMY–2_ gene, one isolate carried an IncI1 plasmid encoding the ARGs *bla*_OXA–1_, *sul1* and *aadA1* and an IncI1/IncFII plasmid which did not carry any ARGs. One isolate carried three plasmids, an IncI1 plasmid encoding the *bla*_CMY–2_ gene, an IncY plasmid and an IncFIA/IncQ1 which harboured 12 ARGs including *bla*_CTX–M–1_ (Table 5). No plasmids were identified from the assembled sequence data of DF0183g, instead the *bla*_CTX–M–15_ gene was chromosomally encoded. All plasmids contained a relaxase and mate-pair formation marker sequences and were therefore classified as conjugative except for plasmid pDF0059.2e_2 which belonged to the IncY group and was non-mobilisable.

**TABLE 5:** Characteristics of plasmids from *E. coli* sequenced with short-read Illumina and long-read MinION methods.

Isolate	Plasmid	Size (bp)	pMLST	Inc. group	CDS*^a^*	ARGs*^b^*	Partial ARGs*^c^*
DF0049.2e	pDF0049.2e_1	89,859	23	IncI1	102	*bla*_*CMY–*2_	None
DF0059.2e	pDF0059.2e_1	94,357	23	IncI1	109	*bla*_*CMY–*2_	None
DF0059.2e	pDF0059.2e_2	97,734	–	IncY	108	None	None
DF0059.2e	pDF0059.2e_3	244,307	[F-: A8: B-]	IncFIA/IncQ1	277	*aac(3)-IId, aadA5, aph(3*′′*)-Ib, aph(3′)-Ia, aph(6)-Id, catA1, bla*_*CTX–M–*1_, *dfrA17, mph(A), sul1, sul2, tet(B)*	*aadA1* (17.2%, 96.0%), *catB4* (19.5%, 100%), *bla*_*TEM–*105_ (31.8%, 100%)
DF0102.4g	pDF0102.4g_1	97,225	26	IncI1	109	*bla*_*OXA–*1_, *sul1, aadA1*	*aadA1* (17.4%, 97.1%), *catB4* (19.5%, 100%)
DF0102.4g	pDF0102.4g_2	58,773	[F35: A-: B-]	IncI1/IncFII	64	None	None
DF0159.2g	pDF0159.2g_1	89,920	23	IncI1	101	*bla*_*CMY–*2_	None
DF0181.1c	pDF0181.1c_1	90,151	23	IncI1	103	*bla*_*CMY–*2_	None
DF0183e	pDF0183e_1	92,691	23	IncI1	106	*bla*_*CMY–*2_	None

^a^CDS, coding sequence. ^b^ARGs, antimicrobial resistance genes defined in Supplementary Table 10. ^c^Partial ARGs, ARGs in which the coverage or identity is less than 80%. Numbers in brackets represent the percentage of coverage and identity, respectively. Inc. group, Plasmid incompatibility group.

DF0059.2e carried an IncFIA/IncQ1 plasmid (244,307 bp) of unknown pMLST for which the nearest plasmid neighbour was the *E. coli* strain T23 multi-drug resistant plasmid pEQ1 (Genbank accession: KF362121). This plasmid carried multiple ARGs (*n* = 12), potentially conferring resistance to aminoglycoside, β-lactam, phenicol, trimethoprim, macrolide, sulfonamide and tetracycline antibiotic classes (Table 5). Physical linkage of ARGs and the location of mobile genetic elements on pDF0059.2e_3 is shown in Supplementary Figure 1. In addition, a partial copy of the *bla*_TEM–105_ (279 bp; 31.82% coverage), the *catB4* (106 bp; 19.49% coverage), and the *aadA1* (166 bp; 17.18% coverage) genes were detected. However, the partial copy of the *catB4* and *aadA1* genes could not be confirmed by visual inspection of the annotated plasmid. The genetic region surrounding the partial *bla*_TEM–105_ gene and the upstream IS26 transposase was extracted (2,438 bp) from the pDF0059.2e_3 plasmid and aligned to the complete *bla*_TEM–105_ reference gene sequence (Genbank accession number: NG_050150, 966 bp). Pairwise alignment showed that 274 of 279 bp of the partial *bla*_TEM–105_ gene was an identical match to the distal end of the reference gene sequence, with the IS26 transposase disrupting the upstream region of the *bla*_TEM–105_ gene (Supplementary Figure 2). We can hypothesise that plasmid pDF0059.2e_3 once harboured a complete copy of the *bla*_TEM–105_ gene and a recombination event involving IS26 transposase disrupted this gene sequence.

Five strains carried IncI1 plasmids (also called IncIα) that were pMLST 23, had a similar number of coding sequences (101–109), encoded the *bla*_CMY–2_ gene and ranged in size from 89,859 to 94,357 bp. Using the MOB-suite database (consisting of 17,779 complete plasmids) the most similar reference to the IncI1 plasmids identified using Mash distances, was the *Salmonella enterica* subspecies enterica serovar Newport plasmid pCVM22462 (Genbank accession number: CP009566.1). Core genome SNP analysis of these five plasmids, using pDF0049.2e_1 as the reference, suggested they were genetically similar. There were no SNP differences between the plasmid-core genome of DF0181.1c and DF0183e, which were isolated from composite faeces and FDE respectively on Dairy 4 in December 2019 (Supplementary Table 5). The core chromosomal genome of these isolates was also genetically similar, differing by only 65 SNPs. *E. coli* DF0181.3c (only short-read sequencing data available) was isolated from the same sampling month as DF0181.1c and DF0183e and had an identical serotype and resistance profile. IncI1 plasmid incompatibility factors were detected in the draft genome for DF0181.3c, and therefore this isolate may carry a similar plasmid as the aforementioned strains, however, this plasmid could not be reconstructed and compared in detail in the absence of long-read sequencing data. Plasmid DF0049.2e_1 was annotated as a representative of the IncI1 plasmids showing the location of the *bla*_CMY–2_ gene, mobile genetic elements and the *tra* genes involved in the conjugal transfer system (Supplementary Figure 3). All plasmids showed variation near the shufflon protein, a genetic region involved in conjugation of IncI1 plasmids (Brouwer et al., 2019).

Two chromosomal mediated AmpC-producing *E. coli* (DF0031.1c and DF0047c) underwent Illumina short-read sequencing only and additional ARGs which potentially confer resistance to aminoglycoside, trimethoprim, sulfonamide and tetracycline antibiotics (trimethoprim and sulfonamide resistance was not phenotypically confirmed) as well as plasmid incompatibility factors were identified. These findings suggest that these isolates harbour additional ARGs, some of which are likely encoded on plasmids due to the co-location of ARGs [*aph(3*′′*)-Ib*, *aph(6)-Id*, *sul2*] and plasmid incompatibility factors (IncQ1) on the same contig and therefore could potentially spread via HGT.

Using VirulenceFinder, the five IncI1 plasmids harboured virulence genes encoding colicins, namely *cia* (*n* = 4, *bla*_CMY–2_ positive plasmids) and *cib* (*n* = 1, pDF0102.4g_1). Plasmid pDF0102.4g_2 harboured the *traT* gene, which encodes an outer membrane protein involved in complement resistance, although the *traT* gene is also a part of the transfer operon in conjugative plasmids and is involved in surface exclusion between identical or closely related plasmids by preventing stable mating aggregates (Sukupolvi and O’Connor, 1990). Plasmids pDF0059.2e_2 and pDF0059.2e_3 did not carry any known virulence genes.

## Discussion

Overall, the sample level prevalence of ESBL-producing *E. coli* from faeces and FDE was low on Dairy 4 and they were not detected on Dairy 1 (Table 1). These results are consistent with a previous regional-based cross-sectional study of NZ dairy farms, which found a low prevalence of ESBL-producing *E. coli* in pooled faecal samples and no *E. coli* with pAmpC genes were identified (Burgess et al., 2021a). Similarly, a nationwide cross-sectional study on dairy farms did not detect any ESBL- or plasmid-mediated AmpC-producing *E. coli* (Burgess et al., 2021b). Overseas studies have found a higher prevalence of AmpC- and/or ESBL-producing *E. coli* from dairy farms, with herd level prevalence estimates ranging from 13% on organic dairy farms in the Netherlands (Santman-Berends et al., 2017) and 5.2–86.7% on conventional farms (Ohnishi et al., 2013; Schmid et al., 2013; Dahms et al., 2015; Gonggrijp et al., 2016; Heuvelink et al., 2019; Schubert et al., 2021).

The sample level prevalence of AmpC-producing *E. coli* in this study was also relatively low (Table 1). AmpC-producing *E. coli* were isolated after months of both high and low AMU, highlighting that additional factors other than total AMU may also play a role in the development and transmission of antimicrobial resistant bacteria in the dairy farm environment. *E. coli* with pAmpC genes were infrequently identified on Dairy 1 (1 of 18; 5.6%) and all AmpC-producing *E. coli* isolated from Dairy 4 were plasmid-mediated (27 of 27; 100%; *bla*_CMY–2_). This suggests that although Dairy 1 had a higher sample level prevalence of AmpC-producing *E. coli*, particularly in FDE, these putative AmpC hyperproducers predominantly spread by vertical transmission. The differences in sample prevalence from FDE may be due to different effluent management strategies between farms, with Dairy 4 storing FDE in a large pond prior to spraying onto paddocks which likely results in the dilution of bacteria. Univariable analysis of data obtained from British beef farms found that spreading of farm manure was significantly associated with an animal testing positive for AmpC-producing *E. coli* (Velasova et al., 2019). A higher number of 3GC resistant *E. coli* were isolated from Dairy 4 in December 2019, which may have been due to several farm management factors including increased AMU during calving and mating. However, there was no increase in 3GC and 4GC use on Dairy 4 during this time period, which has been associated with ESBL/AmpC positive herd status (Gonggrijp et al., 2016). Climatic conditions such as increased temperatures in December, may also play a role. A recent study suggested that lower monthly ambient temperatures were associated with a lower rate of identifying *bla*_CTX–M_ positive *E. coli* samples (Schubert et al., 2021). However, the small number of samples (*n* = 2) positive for ESBL-producing *E. coli* in this study does not allow for any associations between seasonality and ESBL positive samples to be observed. December 2019 was at the end of the study sampling period and no further sampling was undertaken to assess whether this increased trend of 3GC resistant *E. coli* continued in the following months, but no comparable increase was observed during the same 2018 months. However, these observations and climatic factors may assist with future sampling strategies for investigating the prevalence of 3GC *E. coli* on pasture-based dairy farms.

Of the AmpC-producing *E. coli* isolated during this study, 18 of 46 (39.1%) were AmpC hyperproducers. Variable proportions of AmpC hyperproducers have been detected in previous studies, with a small proportion of extended-spectrum cephalosporin resistant *E. coli* isolated from livestock in the Netherlands being AmpC hyperproducers (217 of 2,034; 9.4%) (Ceccarelli et al., 2019) compared to a higher proportion (46.2% of cefotaxime-resistant *E. coli*) that were identified across 53 farms in the United Kingdom (Alzayn et al., 2020; Schubert et al., 2021). Higher use of amoxicillin/clavulanate and sampling of faecal samples from the environment of young calves has been associated with an increased risk of identifying putative *ampC* hyperproducers on dairy farms (Alzayn et al., 2020) and *in vitro* studies have shown an association between amoxicillin use and AmpC-producing *E. coli* arising from mutations in the promoter region of the *ampC* gene (Händel et al., 2014; Stohr et al., 2020). Amoxicillin and clavulanic acid are classified as yellow tier antimicrobials by the NZVA, indicating their use should be restricted in NZ veterinary practices (Anonymous, 2018). Amoxicillin and clavulanic acid were not used on Dairy 1 and Dairy 4 during the study period. Cephalosporin use (including 3GCs) was not identified as a risk factor for putative *ampC* hyperproducing *E. coli* (Alzayn et al., 2020) and 3GC use on Dairy 1 and Dairy 4 was very low during the 15-month study period (<1% total mg/PCU). The lack of known risk factors for AmpC hyperproducers on Dairy 1 highlights the complexity of factors involved in the development of AMR and suggests additional studies are required to identify risk factors for AmpC hyperproducers, particularly in pasture-based dairy farms.

Infections caused by ESBL/AmpC positive *Enterobacterales* pose significant treatment option challenges for clinicians, with cefepime (a 4GC) or carbapenems being suggested as the main treatment options (Meini et al., 2019), both of which are critically important antimicrobials for human medicine (Collignon et al., 2016). One *E. coli* isolated from faeces in this study (1.9%, 1 of 52) was both AmpC and ESBL positive, carrying the *bla*_CMY–2_ and *bla*_CTX–M–1_ genes on two distinct plasmids (Table 4). A low proportion of *E. coli* isolates displayed an ESBL/AmpC phenotype in a study of dairy and beef cattle and sheep farms in Spain (5.2%, 7 of 135 isolates) (Tello et al., 2020). A cross-sectional study of dairy farms in Canada (*n* = 102) also found a low proportion of ESBL/AmpC positive *E. coli* (2%) in comparison to AmpC (51%) and ESBL (46%) phenotypes (Massé et al., 2021). These findings suggest that *E. coli* displaying both an ESBL and AmpC phenotype are infrequently isolated from dairy farm environments.

ESBL- and AmpC-producing *E. coli* were not detected in soil from recently grazed paddocks or bulk tank milk samples on either farm. Despite a small sample size, the lack of detection from these matrices over a 15-month period indicates that they may be potentially uncommon sources of ESBL- and AmpC-producing *E. coli* in the NZ dairy farm environment, whereas faeces and FDE are more probable sources. At the sample level, *bla*_CTX–M_ or *bla*_CMY–2_ positive *E. coli* were infrequently detected from soil samples across 17 commercial beef farms in the United States (3.89%; 3 of 77) (Lee et al., 2020) and the prevalence of ESBL- and AmpC-producing *Enterobacteriaceae* from bulk tank milk was more varied, ranging from 0 to 9.5% (Geser et al., 2012; Skockova et al., 2015; Sudarwanto et al., 2015; Odenthal et al., 2016). The lack of detection of ESBL-producing *E. coli* in bulk tank milk in NZ is not unexpected due to the stringent hygiene and food safety standards for dairy farming and milk storage in NZ (Fonterra Co-operative Group Limited, 2018).

It is important to consider variations in study design (sample size and animal age/health status), sample matrices and culture selection methods when comparing between prevalence studies. For example, this study did not use a pre-enrichment step for ESBL- and AmpC-producing *E. coli* prior to plating on selective agar, which is a technique used in some prevalence studies (Hordijk et al., 2013; Gonggrijp et al., 2016; Hutchinson et al., 2017). In addition, selecting one isolate per sample may not be sufficient to reflect the bacterial heterogeneity associated with an ecological niche (Venturini et al., 2022), therefore, analysing multiple isolates per sample is beneficial in prevalence studies. The age of the study population is also a crucial factor to consider. For example, a study of 101 dairy farms in Canada detected ESBL- or AmpC-producing *E. coli* at least once during the study in 85% of farms, although the majority were isolates from calves (Massé et al., 2021). A longitudinal study of a United Kingdom dairy farm in which CTX-M-15 ESBL *E. coli* had previously been identified from a septic neonatal calf, found a higher proportion of *E. coli* positive for the *bla*_CTX–M_ gene in milking cows (30.3%) compared with non-milking cows (3.0%) (Watson et al., 2012), although this study did not look for other ESBL enzyme types and other management factors (e.g., AMU) differed between cattle groups.

Variation in farming systems between countries is also important to consider when comparing studies. Intensive farming systems, particularly indoor housing, have been associated with a higher prevalence of mastitis (Lacy-Hulbert et al., 2002), which can lead to higher AMU and subsequently increased levels of AMR. It has been proposed that the NZ pasture-based farming system, in conjunction with low AMU in food-producing animals, may contribute to lower levels of AMR (Collis et al., 2019). The low sample level prevalence of ESBL- and AmpC-producing *E. coli* from Dairy 1 and Dairy 4 supported this hypothesis. Factors linked to less intensive farming practices have been associated with fewer samples positive for cefotaxime resistant *E. coli* samples from both beef and dairy cattle (Hille et al., 2017). A cross-sectional study of grazing beef cattle farms (*n* = 17) in the United States found that larger farming operations (>500 cattle) were associated with a 58% higher likelihood of detecting cefotaxime resistant bacteria from faecal samples (Markland et al., 2019).

Both farms in this present study operated a closed herd system (no introduction of off-farm animals), which may reduce the risk of introductions of antimicrobial resistant bacteria into the herd from outside cattle sources. For example, on British beef farms, buying bulls or fattening cattle have been identified as risk factors for *bla*_CTX–M_ positive *Enterobacteriaceae* or AmpC-producing *E. coli*, respectively (Velasova et al., 2019). However, the impact that introductions of antimicrobial resistant bacteria has compared to AMR selection in agricultural environments is unknown.

AMU on farms is influenced by a number of management factors including average age of the herd, disease outbreaks, hygiene practices, the use of teat sealants as well as the farmers perception toward antimicrobial stewardship. Interestingly, Dairy 1 had a higher total AMU during the study period, yet no ESBL-producing *E. coli* were detected on Dairy 1 (Supplementary Tables 8, 9). However, a higher proportion of the total AMU on Dairy 1 was classified as green tier antimicrobials by the NZVA with Dairy 4 using more of yellow tier antimicrobials. The variation, predominantly green on Dairy 1 and yellow tier classes on Dairy 4, is likely associated with the predominant illnesses treated per farm. Other factors may have an impact on the AMU on farms, such as the average age of the cows, since the incidence of clinical mastitis is higher in older cows (Petrovski et al., 2009). The estimated base rate for use of antimicrobials in food-producing animals in NZ in 2018 was 10.21 mg/PCU (Hillerton et al., 2021). The representative total AMU during the study period on Dairy 1 and Dairy 4 was higher (17.09 mg/PCU) and lower (5.36 mg/PCU) than this estimate, respectively. The AMU on Dairy 1 and Dairy 4 was also within the range reported in a cross-sectional study of 26 dairy farms across NZ (4.39 – 20.92 mg/PCU) (Burgess et al., 2021b), albeit at the higher and lower end of the spectrum, respectively. The total AMU was estimated using sales data in the aforementioned study, whereas individual antimicrobial treatments were used for calculating the total AMU in this study, which makes comparisons difficult. The use of 3GCs (<1% total mg/PCU), which have been identified as a risk factor for ESBL-producing *Enterobacteriaceae* (Gonggrijp et al., 2016), and use of NZVA red tier classified antimicrobials.

The ESBL- and AmpC-producing *E. coli* which were sequenced in this study belonged to a diverse range of STs and serotypes. Similar findings have been reported for *bla*_CMY–2_ positive *E. coli* from human clinical cases, livestock and food matrices in which a diverse range of STs were reported (Pietsch et al., 2018). In contrast, *E. coli* ST131, which are frequently multi-drug resistant and harbour *bla*_CTX–M_ genes, are widely disseminated in humans globally (Banerjee and Johnson, 2014; Jafari et al., 2020). The two sequenced AmpC hyperproducers belonged to ST88 (phylogroup C), which is consistent with previous findings that AmpC hyperproducers predominantly belong to this sequence type (Haenni et al., 2014b; Alzayn et al., 2020). In contrast, three AmpC hyperproducing *E. coli* previously isolated from dairy cattle in NZ belonged to ST1148 (*n* = 2) and ST298 (*n* = 1) (Burgess et al., 2021a). ESBL-producing *E. coli* were isolated from two samples (faeces and FDE) in this study. Isolate DF0183g, belonging to ST4553, harboured a chromosomally encoded *bla*_CTX–M–15_. ST4553 *E. coli* positive for the *bla*_CTX–M–15_ gene have also been detected in dog faeces (*n* = 1) (Toombs-Ruane et al., 2021) and storm water (*n* = 3) in NZ (Burgess et al., 2021c). *E. coli* DF0059.2e belongs to ST5135 and to the best of our knowledge, this is the first *E. coli* ST5135 *bla*_CTX–M–1_ to be reported. Few *E. coli* ST5135 have been reported in Enterobase Zhou et al., 2020, and those identified were isolated from human, livestock, canine and poultry samples (accessed 23rd May 2022).

The ESAC production was examined for DF0102.4g and any ESBL negative *E. coli* isolated from CHROMagar™ ESBL plates (DF0159.2g). SNPs previously identified in ESAC producers (Santiago et al., 2018) were detected in the areas coding for the omega loop region for both DF0102.4g and DF0159.2g (K191Q and S209P). These SNPs result in AA substitutions with different biochemical characteristics, which may result in modifications in the omega loop (Santiago et al., 2018). However, these AA substitutions have also been described in *E. coli* which were susceptible to cefepime, and therefore unlikely to be ESAC producers (Burgess et al., 2021b). Isolate DF0102.4g had a further substitution in the omega loop (N201T), but this resulted in substitution of an AA with the same charge which is likely to have a minimal impact on the secondary, tertiary and quaternary structure of the enzyme. DF0159.2g contained a SNP leading to an AA substitution (I300V) in the R2 loop region, which is part of the catalytic site of AmpC and substitutions in this region may result in conformational changes and flexibility in the hydrolysis spectrum of AmpC (Santiago et al., 2018). Due to the combination of AA substitutions in DF0159.2g in both the omega and R2 loops, we hypothesise that this isolate is potentially an ESAC producer. ESAC production could not be inferred for isolate DF0102.4g due to the absence of AA substitutions in the R2 loop region (Table 2), however, this isolate had AA substitutions (E140D, N201T, N260T, and W370C), which to the best of our knowledge, have not previously been reported (Haenni et al., 2014a; Santiago et al., 2018; Burgess et al., 2021b) and the role these may have on the AmpC β-lactamase spectrum of activity are unknown.

*E. coli* DF0102.4g underwent whole genome sequencing using both Illumina and MinION sequencing methods and carried a plasmid which harboured the *bla*_OXA–1_, *sul1*, and *aadA1* genes as well as partial copies of the *aadA1* and *catB4* gene (Table 4). The *bla*_OXA–1_ gene encodes a narrow-spectrum β-lactamase which traditionally confers resistance to aminopenicillins, carboxypenicillins and ureidopenicllins (Torres et al., 2015). However, overexpression of this gene has been shown to confer reduced susceptibility to 4GCs such as cefepime and susceptibility to cefotaxime and ceftazidime when coupled with porin loss (OmpC and/or OmpF) (Beceiro et al., 2011). Three AmpC-producing *E. coli*, with mutations in the promoter region of the *ampC* gene, isolated from United Kingdom dairy farms also carried the *bla*_OXA–1_ gene and showed reduced susceptibility to cefepime (Alzayn et al., 2020). Possible resistance mechanisms of *E. coli* DF0102.4g to 3GC and 4GC warranting further investigation include the overexpression of the *bla*_OXA–1_ gene coupled with porin loss assessed using expression studies and proteomics, respectively or modification and mutations in other genes such as the *mar* genes.

Plasmids play a major role in the dissemination of AMR in *Enterobacteriaceae* (Rozwandowicz et al., 2018). The detection of ARGs on plasmids is of particular concern due to the potential for dissemination within bacterial populations, particularly to pathogenic bacteria. ESBL genes are often encoded on plasmids that carry several ARGs, resulting in a multi-drug resistant phenotype (Ho et al., 2013; McGann et al., 2016). Isolate DF0059.2e (ST5135) harboured three plasmids, two of which encoded ARGs including pDF0059.2e_2 which carried the *bla*_CMY–2_ gene. pDF0059.2e_1 belonged to the IncY group, which are prophages that autonomously replicate and have been reported to be co-associated with other plasmid types including IncF and/or IncI (Rozwandowicz et al., 2018), as was seen in this isolate. Although no ARGs were encoded on plasmid pDF0059.2e_1, IncY plasmids have been reported to carry resistance genes including *bla*_CTX–M–15_ in *K. pneumoniae* (Ribeiro et al., 2016) and *mcr-1* in an *E. coli* isolated from a pig farm in China (Chunping et al., 2021). The third plasmid, pDF0059.2e_3, harboured 12 ARGs including *bla*_CTX–M–1_ which were all physically linked. Detection of mobile genetic elements surrounding the ARGs suggests that this plasmid has undergone recombination events, potentially contributing to the development of a multi-drug resistant plasmid in this isolate.

Plasmid associated β-lactamase gene types have been shown to have a highly homologous genetic environment, regardless of source (Lee et al., 2020). In this study, five strains harboured highly similar IncI1 plasmids which encoded the *bla*_CMY–2_ gene. IncI plasmids are conjugative, have a low copy number and a narrow host range (Rozwandowicz et al., 2018). A high proportion of ESBL and AmpC genetic determinants have also been found on IncI1 plasmids in other studies (Tello et al., 2020; Ewers et al., 2021), with IncI1 plasmids being frequently identified from extended-spectrum cephalosporin resistant *E. coli* isolated from healthy livestock in the Netherlands over a 10 years period (Ceccarelli et al., 2019). However, the plasmid and ESBL/AmpC gene combinations in *E. coli* isolated from dairy cattle and veal calves was more variable compared to the plasmid and gene combinations identified in *E. coli* isolated from other livestock sectors (Ceccarelli et al., 2019). A diverse range of STs and phylogenetic groups were detected among 3GC resistant *Escherichia* spp. isolated from healthy cattle, pigs and chickens. This suggested that the clonal spread of single lineages is unlikely to have occurred (Ewers et al., 2021). Together, these data and this study highlight the importance of horizontal plasmid transfer in the dissemination of 3GC resistance among *Enterobacteriaceae*.

## Conclusion

This study assessed the sample level prevalence of ESBL- and AmpC-producing *E. coli* on two NZ dairy farms over a 15-month period. No ESBL or AmpC-producing *E. coli* were isolated from bulk tank milk or soil from recently grazed paddocks in this study, suggesting they are less likely reservoirs in the NZ dairy farm environment compared with faeces and FDE. ESBL-producing *E. coli* were infrequently identified during the study, suggesting they are present at a low prevalence in these two dairy farms. Plasmid-mediated AmpC-producing *E. coli* were isolated at a low prevalence in faeces and FDE but were isolated at a higher frequency on the larger farm (Dairy 4) compared with Dairy 1. In contrast, AmpC-producing *E. coli* with mutations in the promoter region of the chromosomal *ampC* gene were detected at a higher prevalence, particularly in FDE but only on Dairy 1. The detection of AmpC-producing *E. coli* with mutations in the promoter region of the *ampC* gene is less concerning in regard to the dissemination of AMR within bacterial populations. These findings highlight the necessity to confirm the genotype of an AmpC phenotype in *E. coli*.

Both ESBL- and AmpC-producing *E. coli* were isolated at various time points throughout the 15-month study period and their detection was not associated with periods of elevated AMU on NZ dairy farms. Dairy 1 had higher AMU (mg/PCU) than Dairy 4 during this study period, however, both farms used a low proportion of 3GC and 4GCs which are known risk factors for ESBL-producing *Enterobacteriaceae*. In addition, the farms used low levels of antimicrobials classed as red tier by the NZVA and these results support the hypothesis that low AMU on NZ dairy farms influences the prevalence of AMR in the dairy farm environment. Additional studies should focus on high-risk animals such as young calves and a larger number of dairy farms throughout NZ, including farms with known risk factors or management practices which may influence AMR such as high use of 3GC and 4GCs, an open herd system, feeding waste milk to calves and the use of blanket dry cow therapy.

## Data availability statement

The BioProject PRJNA844174 and BioSample accession numbers have been included in Supplementary Table 5.

## Author contributions

RC, PB, SB, AM, GB, and AC involved in the project conceptualisation. RC, AC, and GB acquired project funding. RC, PB, SB, AM, and AC conducted investigation, formal analysis, and methodology development. AC and GB involved in the project administration. RC, PB, SB, AM, GB, and AC wrote the manuscript. All authors contributed to the manuscript revision, read, and approved the submitted version.
