# Supplementary material for: Prevalence and distribution of extended-spectrum β-lactamase and AmpC-producing Escherichia coli in two New Zealand dairy farm environments
Source: Front Microbiol. 2022 Aug 11;13:960748. doi: 10.3389/fmicb.2022.960748 (PMC9403332; doi:10.3389/fmicb.2022.960748)
Supplement: Supplementary file 1 [file Table_1.DOCX]

Table S1 A comparison of farm parameters and management practices between the two farms recruited in this study.

| **Parameter** | **Dairy 1^a^** | **Dairy 4** |
| --- | --- | --- |
| Size (hectares) | 142.7 | 250 |
| Number of cows during the study period | 261 | 584 |
| Predominant breed | Friesian, Jersey and Friesian/Jersey cross | Friesian, Jersey and Friesian/Jersey cross |
| Milking frequency | Once a day | Morning and afternoon |
| Effluent management strategy | The FDE management strategy on Dairy 1 changed during the study period. From October 2018, the FDE was filtered using the dairy effluent recycling system by Forsi Innovations and applied to paddocks. Due to technical issues, from July 2019 onwards the FDE was not applied to paddocks and was discharged into the sewage system. | FDE is applied to the paddocks which is a common practice on NZ dairy farms. |

a FDE, farm dairy effluent
