# Supplementary material for: Prevalence and distribution of extended-spectrum β-lactamase and AmpC-producing Escherichia coli in two New Zealand dairy farm environments
Source: Front Microbiol. 2022 Aug 11;13:960748. doi: 10.3389/fmicb.2022.960748 (PMC9403332; doi:10.3389/fmicb.2022.960748)
Supplement: Supplementary file 2 [file Table_2.DOCX]

Table S2: Sample collection and processing methods.

| **Sample^a^** | **Source** | **Collection method** | **Processing steps** | **Sample volume or weight** | **Buffered peptone water**  **(mL)** |
| --- | --- | --- | --- | --- | --- |
| Pooled faeces | Recently grazed paddock (grazed the night prior to sampling). | Sixteen fresh faecal samples randomly collected from the paddock and pooled as groups of four. | The prevalence of ESBL-producing *E. coli* was expected to be low, therefore four faecal samples were pooled. A pea-size amount of each faecal sample was transferred using a sterile cotton swab into buffered peptone water. | Pea-size amount per sample | 15 |
| FDE | Dairy 1, two sample collection points were used. Collection point one was the effluent sump by the cow-shed (October 2018 - May 2019) and collection point two was from a grate in the cow-shed (July 2019 - December 2019). For Dairy 4, the FDE was collected from the effluent pond. | Approximately 500 mL FDE collected in two sterile 250 mL Schott bottles attached to the end of a pole. | Approximately 400 mL of FDE was centrifuged at 10,000 x *g* (Sorvall LYNX 4000 Superspeed Centrifuge, Thermo Fisher Scientific, Waltham, Massachusetts, United States) for 20 min at 4°C. After, the supernatant was decanted and the pellet was re-suspended in 2 ml of the reserved supernatant. | 100 µL re-  suspended pellet (equates to approximately 20 mL of FDE) | 9.9 |
| Soil | Recently grazed paddock (grazed the night prior to sampling). | Four soil cores (10 cm depth; 3 cm diameter) collected from near the centre of the paddock and were pooled to form one composite sample. Each soil core was taken in a transect line, approximately 1 m apart. The collection of pasture and faeces was avoided. | Soil samples homogenised by hand in the WhirlPak bag. | 1 g | 9 |
| Milk | Milk vat | Approximately 1 L collected from the milking vat. On Dairy 4, milk collected from the morning milking. | Approximately 400 mL of milk was centrifuged at 10,000 x *g* (Sorvall LYNX 4000 Superspeed Centrifuge, Thermo Fisher Scientific, Waltham, Massachusetts, United States) for 45 min at 4°C. The fat was scooped off and the supernatant was decanted. The pellet was re-suspended in 2 ml of the reserved supernatant. | 100 µL re- suspended pellet (equates to approximately 20 mL of bulk tank milk) | 9.9 |

^a^ FDE, Farm dairy effluent
