# Supplementary material for: Prevalence and distribution of extended-spectrum β-lactamase and AmpC-producing Escherichia coli in two New Zealand dairy farm environments
Source: Front Microbiol. 2022 Aug 11;13:960748. doi: 10.3389/fmicb.2022.960748 (PMC9403332; doi:10.3389/fmicb.2022.960748)
Supplement: Supplementary file 4 [file Table_4.DOCX]

| Table S4: PCR reaction and gel conditions | | | | | |
| --- | --- | --- | --- | --- | --- |
| **PCR reaction** | **Primer set(s)** | **Reaction conditions** | **Gel conditions** | **Controls** | **Ref** |
| *ampC* promoter region | AmpC^171^/AmpC2^120^ | Initial denaturation at 94°C for 90 sec, then 30 cycles of 94°C for 30 sec, 57°C for 30 sec, and 72°C for 1 min, with a final extension step at 72°C for 10 min. | 2% agarose gel run at 90V for 40 min. | LT9031a (positive *E. coli*), FS192 (negative *Staphylococcus aureus*) and no template control. | [1] |
| pAmpC | MOXMF/MOXMR  CITMF/CITMR  DHAMF/DHAMR ACCMF/ACCMR  FOXMF/FOXMR  CMY-F/CMY-R | Initial denaturation at 94°C for 3 min, then 25 cycles of 94°C for 30 sec, 64°C for 30 sec, and 72°C for 1 min, with a final extension step at 72°C for 7 min. | 2% agarose gel run at 80V for 90 min. | NZRM4402 (CMY-positive *E. coli*), NZRM4464 (FOX-5-positive *K. pneumoniae*), NZRM4403 (DHA-1-positive *E. coli*), ATCC25922 (negative *E. coli*) and no template control. | [2] |
| CMY group | CMY-2-F/CMY-2-R | Initial denaturation at 94°C for 3 min, then 30 cycles of 94°C for 30 sec, 58°C for 30 sec, and 72°C for 1 min, with a final extension step at 72°C for 10 min. | 2% agarose gel run at 80V for 40 min. | NZRM4402 (CMY-positive *E. coli*), ATCC25922 (negative *E. coli*) and no template control. | [3] |
| CTX-M-1 | CTX-1-SEQ-F/CTX-1-SEQ-R | Initial denaturation at 95°C for 5 min, then 30 cycles of 95°C for 30 sec, 60°C for 30 sec, and 72°C for 1 min, with a final extension step at 72°C for 1 min. | 0.8% agarose gel run at 80V for 40 min. | DF0183g (CTX-M-15 positive *E. coli*), DF0025d (CTX-M negative *E. coli*) and no template control. | [3] |
| Clermont quadruplex PCR typing method | chuA.1b/chuA.2 yjaA.1b/yjaA.2b  TspE4C2.1b/ TspE4C2.2b AceK.f/ArpA1.r | The PCR was undertaken in a Bio-Rad T100 Thermal Cycler (Bio-Rad, USA) using KAPA HiFi HotStart ReadyMix (KAPA BioSystems, Wilmington, USA) under the following conditions: initial denaturation at 94°C for 4 min, then 30 cycles of 98°C for 20 sec, 61°C for 20 sec, and 72°C for 5 min. | 2% agarose gel run at 80V for 90 min. | AGR3560 (Clade III, IV or V positive *E. coli*), mixed *E. coli* DNA positive for *arpA, chuA, yjaA* and TspE4C2 PCR products and no template control. | [4, 5] |
| Clermont typing method: Group C | trpAgpC.1/ trpAgpC.2 | PCR was undertaken in a Bio-Rad T100 Thermal Cycler (Bio-Rad, USA) using KAPA HiFi HotStart ReadyMix (KAPA BioSystems, Wilmington, USA) under the following conditions: initial denaturation at 94°C for 4 min, then 30 cycles of 98°C for 20 sec, 58°C for 20 sec, and 72°C for 5 min. | 2% agarose gel run at 80V for 90 min. | AGR4288 (*E. coli* phylogroup C positive control) and no template control. | [6] |
| Clermont typing method: Group E | ArpAgpE.f/ ArpAgpE.r | PCR was undertaken in a Bio-Rad T100 Thermal Cycler (Bio-Rad, USA) using KAPA HiFi HotStart ReadyMix (KAPA BioSystems, Wilmington, USA) under the following conditions: initial denaturation at 94°C for 4 min, then 30 cycles of 98°C for 20 sec, 58°C for 20 sec, and 72°C for 5 min. | 2% agarose gel run at 80V for 90 min. | *E. coli* O157 (phylogroup E positive control) and no template control. | [6] |

PCR primers

| **PCR reaction** | **Target** | **Primer set** | **Primer sequence (5' to 3')** | **Product size (bp)** | **Reference** |
| --- | --- | --- | --- | --- | --- |
| AmpC promoter region | Chromosomal *ampC* | AmpC171 AmpC2120 | AATGGGTTTTCTACGGTCTG GGGCAGCAAATGTGGAGCAA | 191 | [1] |
| pAmpC | MOX-1, MOX-2, CMY-1, CMY-8 to CMY-11 | MOXMF MOXMR | GCTGCTCAAGGAGCACAGGAT CACATTGACATAGGTGTGGTG | 520 | [2] |
| pAmpC | LAT-1 to LAT-4, CMY-2 to CMY-7, BIL-1 | CITMF  CITMR | TGGCCAGAACTGACAGGCAAA TTTCTCCTGAACGTGGCTGGC | 462 |  |
| pAmpC | DHA-1, DHA-2 | DHAMF  DHAMR | CCGTACGCATACTGGCTTTGC AACAGCCTCAGCAGCCGGTTA | 405 |  |
| pAmpC | ACC | ACCMF  ACCMR | AACAGCCTCAGCAGCCGGTTA TTCGCCGCAATCATCCCTAGC | 346 |  |
| pAmpC | FOX-1 to FOX-5b | FOXMF  FOXMR | CAAAGCGCGTAACCGGATTGG AACATGGGGTATCAGGGAGATG | 190 |  |
| CMY-group | CMY-group | CMY-2-F  CMY-2-R | ATGATGAAAAAATCGTTATGCTGC GCTTTTCAAGAATGCGCCAGG | 1,138 | [3] |
| CTX-M-1 | CTX-M-1-group | CTX-1-SEQ-F  CTX-1-SEQ-R | CCCATGGTTAAAAAATCACTGC CAGCGCTTTTGCCGTCTAAG | >1,000 |  |
| Quadruplex | *chuA* | chuA.1b  chuA.2 | ATGGTACCGGACGAACCAAC TGCCGCCAGTACCAAAGACA | 288 | [4, 5] |
| Quadruplex | *yjaA* | yjaA.1b  yjaA.2b | CAAACGTGAAGTGTCAGGAG AATGCGTTCCTCAACCTGTG | 211 | [4] |
| Quadruplex | TspE4.C2 | TspE4C2.1b TspE4C2.2b | CACTATTCGTAAGGTCATCC AGTTTATCGCTGCGGGTCGC | 152 | [4] |
| Quadruplex | *arpA* | AceK.f ArpA1.r | AACGCTATTCGCCAGCTTGC TCTCCCCATACCGTACGCTA | 400 | [5, 7] |
| Group C | *trpA* | trpAgpC.1 trpAgpC.2 | AGTTTTATGCCCAGTGCGAG TCTGCGCCGGTCACGCCC | 219 | [6] |
| Group E | *arpA* | ArpAgpE.f ArpAgpE.r | GATTCCATCTTGTCAAAATATGCC GAAAAGAAAAAGAATTCCCAAGAG | 301 |  |

**Sequencing reactions**

Briefly, separate sequencing reactions were performed in 10µl reaction volumes, using the same forward and reverse primers, with 1.75µl buffer, 0.5µl Big Dye™ Terminator v3.1 (ThermoFisher Scientific Inc., Waltham, MA, USA) and 1µl primer (3.2 pm/µl). For sequencing of the *ampC* gene promoter region PCR product (191 bp), 5.75µl sterile molecular biology-grade water and 1µl DNA template (3 ng) was used. For sequencing of *bla*_CMY-2_ and *bla*_CTX-M-1_ PCR products (1,138 and >1000 bp, respectively), 4.75µl sterile molecular biology-grade water and 2µl DNA template (12 ng) was used. The sequencing PCR reactions were undertaken on a Bio-Rad T100 Thermal Cycler (Bio-Rad, Waltham, MA, USA) using the following conditions: 95°C for 1 min, then 25 cycles of 95°C for 10 sec, 50°C for 10 sec, and 60°C for 90 sec.

**References**

1. Caroff, N., et al., *Mutations in the ampC promoter of Escherichia coli isolates resistant to oxyiminocephalosporins without extended spectrum beta-lactamase production.* FEMS Microbiol Lett, 1999. **173**(2): p. 459-65.

2. Pérez-Pérez, F.J. and N.D. Hanson, *Detection of plasmid-mediated AmpC β-lactamase genes in clinical isolates by using multiplex PCR.* Journal of Clinical Microbiology, 2002. **40**(6): p. 2153-2162.

3. Dierikx, C.M., et al., *Occurrence and characteristics of extended-spectrum-β-lactamase- and AmpC-producing clinical isolates derived from companion animals and horses.* Journal of Antimicrobial Chemotherapy, 2012. **67**(6): p. 1368-1374.

4. Clermont, O., et al., *The Clermont Escherichia coli phylo-typing method revisited: improvement of specificity and detection of new phylo-groups.* Environ Microbiol Rep, 2013. **5**(1): p. 58-65.

5. Clermont, O., S. Bonacorsi, and E. Bingen, *Rapid and simple determination of the <i>Escherichia coli</i> phylogenetic group.* Applied and Environmental Microbiology, 2000. **66**(10): p. 4555-4558.

6. Lescat, M., et al., *Commensal Escherichia coli strains in Guiana reveal a high genetic diversity with host-dependant population structure.* Environmental Microbiology Reports, 2013. **5**(1): p. 49-57.

7. Clermont, O., S. Bonacorsi, and E. Bingen, *Characterization of an anonymous molecular marker strongly linked to <i>Escherichia coli</i> strains causing neonatal meningitis.* Journal of Clinical Microbiology, 2004. **42**(4): p. 1770-1772.
