# Supplementary material for: Prevalence and distribution of extended-spectrum β-lactamase and AmpC-producing Escherichia coli in two New Zealand dairy farm environments
Source: Front Microbiol. 2022 Aug 11;13:960748. doi: 10.3389/fmicb.2022.960748 (PMC9403332; doi:10.3389/fmicb.2022.960748)
Supplement: Supplementary file 7 [file Table_7.docx]

| **Antimicrobial product** | **Amount (mg)** | **Administration route†** | **Active ingredient** | **Class** |
| --- | --- | --- | --- | --- |
| Betamox LA | 6,000 | Parenteral other | Amoxycillin | Aminopenicillins |
| Bivatop® 200 | 20,000 | LCT par | Oxytetracycline | Tetracyclines |
| Bomacure | 20,000 | Parenteral other | Cephapirin | First-generation cephalosporins |
| Metri-Clean™ or Metricure® | 7,000 | Parenteral other | Cephapirin | First-generation cephalosporins |
| Penethaject | 966,570 | Parenteral other | Penethamate | Penicillins |
| Vibrostrep™ | 80,000 | Parenteral other | Streptomycin | Aminoglycosides |
| Orbenin DC | 12,000 | DCT | Cloxacillin | Penicillins |
| Intracillin® 1000 Milking Cow | 336,000 | Parenteral other | Procaine penicillin G | Penicillins |
| Excede LA | 14,000 | LCT par | Ceftiofur | Third-generation cephalosporins |
| Intracillin® 300 | 495,000 | Other | Procaine penicillin G | Penicillins |
| Marbocyl 10% | 1,200 | Other | Marbofloxacin | Quinolones |
| Phoenix Pharmacillin 300 | 22,500 | Other | Procaine penicillin G | Penicillins |
| Forcyl | 40,320 | Other | Marbofloxacin | Quinolones |
| **Total** | 2,020,590 |  |  |  |

**Table S7:** Antimicrobial use on Dairy 1

**Amount (mg) of antimicrobial products used on Dairy 1 during the study period**

†: DCT, dry cow therapy; LCT par, lactating cow therapy parenteral

**Amount (mg) of antimicrobial per class used on Dairy 1**

| **Class** | **Amount (mg)** | **PCU†** | **% of total** |
| --- | --- | --- | --- |
| Aminoglycoside | 80,000 | 0.68 | 3.96 |
| Aminopenicillin | 6,000 | 0.05 | 0.30 |
| First-generation cephalosporin | 27,000 | 0.23 | 1.34 |
| Third-generation cephalosporin | 14,000 | 0.12 | 0.69 |
| Penicillin | 1,832,070 | 15.50 | 90.67 |
| Quinolone | 41,520 | 0.35 | 2.05 |
| Tetracycline | 20,000 | 0.17 | 0.99 |
| **Total** | 2,020,590 | 17.09 | 100.00 |

†: PCU, Population correction unit.

**Antimicrobial use per month on Dairy 1**

| **Date** | **Amount (mg)** | **PCU†** |
| --- | --- | --- |
| October 2018 | 248,782 | 2.10 |
| November 2018 | 93,494 | 0.79 |
| December 2018 | 58,830 | 0.50 |
| January 2019 | 78,496 | 0.66 |
| February 2019 | 97,492 | 0.82 |
| March 2019 | 127,996 | 1.08 |
| April 2019 | 30,500 | 0.26 |
| May 2019 | 21,698 | 0.18 |
| June 2019 | 48,996 | 0.41 |
| July 2019 | 50,898 | 0.43 |
| August 2019 | 376,648 | 3.19 |
| September 2019 | 362,268 | 3.06 |
| October 2019 | 175,656 | 1.49 |
| November 2019 | 183,840 | 1.55 |
| December 2019 | 64,996 | 0.55 |
| **Total** | 2,020,590 | 17.09 |

†: PCU, Population correction unit.
