# Supplementary material for: Prevalence and distribution of extended-spectrum β-lactamase and AmpC-producing Escherichia coli in two New Zealand dairy farm environments
Source: Front Microbiol. 2022 Aug 11;13:960748. doi: 10.3389/fmicb.2022.960748 (PMC9403332; doi:10.3389/fmicb.2022.960748)
Supplement: Supplementary file 8 [file Table_8.docx]

| **Antimicrobial product** | **Amount (mg)** | **Administration route†** | **Antimicrobial ingredient** | **Class** |
| --- | --- | --- | --- | --- |
| Betamox LA | 6,000 | Parenteral other | Amoxycillin | Aminopenicillins |
| Bivatop® 200 | 10,000 | LCT par | Oxytetracycline | Tetracyclines |
| Bomacure | 750,000 | Parenteral other | Cephapirin | First-generation cephalosporins |
| Mastalone® | 49.19 | Parenteral other | Oxytetracycline, oleandomycin, neomycin | Multiple classes |
| Penethaject | 43,329 | Parenteral other | Penethamate | Penicillins |
| Tylo 200 | 5,000 | Parenteral other | Tylosin | Macrolides |
| Vibrostrep™ | 50,000 | Parenteral other | Streptomycin | Aminoglycosides |
| Orbenin DC | 84,000 | DCT | Cloxacillin | Penicillins |
| Intracillin® 1000 Milking Cow | 1,000 | Parenteral other | Procaine penicillin G | Penicillins |
| Penclox 1200™ | 72,000 | Parenteral other | Penicillin G and cloxacillin | Penicillins |
| Excede LA | 10,600 | LCT par | Ceftiofur | Third-generation cephalosporins |
| Intracillin® 300 | 121,800 | Other | Procaine penicillin G | Penicillins |
| Marbocyl 10% | 8,300 | Other | Marbofloxacin | Quinolones |
| Phoenix Pharmacillin 300 | 255,000 | Other | Procaine penicillin G | Penicillins |
| **Total** | 1,417,078.19 |  |  |  |

**Table S8:** Antimicrobial use on Dairy 4

**Amount (mg) of antimicrobial products used on Dairy 4 during the study period**

†: DCT, dry cow therapy; LCT par, lactating cow therapy parenteral

**Amount (mg) of antimicrobial per class used on Dairy 4**

| **Class** | **Amount (mg)** | **PCU†** | **% of total** |
| --- | --- | --- | --- |
| Aminoglycoside | 50,000 | 0.19 | 3.53 |
| Aminopenicillin | 6,000 | 0.02 | 0.42 |
| First-generation cephalosporin | 750,000 | 2.83 | 52.93 |
| Third-generation cephalosporin | 10,600 | 0.04 | 0.75 |
| Penicillin | 577,129 | 2.18 | 40.73 |
| Quinolone | 8,300 | 0.03 | 0.59 |
| Tetracycline | 10,000 | 0.04 | 0.71 |
| Multiple classes | 49.19 | 0.00 | 0.00 |
| Macrolide | 5,000 | 0.02 | 0.35 |
| Total | 1,417,078.19 | 5.36 | 100.00 |

†: PCU, Population correction unit

**Antimicrobial use per month on Dairy 4**

| **Date** | **Amount (mg)** | **PCU†** |
| --- | --- | --- |
| November 2018 | 57,399 | 0.22 |
| December 2018 | 2,400 | 0.01 |
| January 2019 | 44,100 | 0.17 |
| February 2019 | 13,800 | 0.05 |
| March 2019 | 11,200 | 0.04 |
| April 2019 | 42,000 | 0.16 |
| May 2019 | 32,000 | 0.12 |
| June 2019 | 57,000 | 0.22 |
| July 2019 | 149,800 | 0.57 |
| August 2019 | 66,200 | 0.25 |
| September 2019 | 804,447.19 | 3.04 |
| October 2019 | 56,000 | 0.21 |
| November 2019 | 58,632 | 0.22 |
| December 2019 | 22,100 | 0.08 |
| **Total** | 1,417,078.19 | 5.36 |

†: PCU, Population correction unit
