# Supplementary material for: Prevalence and distribution of extended-spectrum β-lactamase and AmpC-producing Escherichia coli in two New Zealand dairy farm environments
Source: Front Microbiol. 2022 Aug 11;13:960748. doi: 10.3389/fmicb.2022.960748 (PMC9403332; doi:10.3389/fmicb.2022.960748)
Supplement: Supplementary file 9 [file Table_9.DOCX]

Table S9 Amount of the antimicrobial classes used on Dairy 1 and Dairy 4 between October 2018 and December 2019

| **Antimicrobial class** | **Dairy 1^a^ (mg/PCU)** | **Dairy 4 (mg/PCU)** |
| --- | --- | --- |
| Aminoglycosides | 0.68 (4.0%) | 0.19 (3.5%) |
| Aminopenicillins | 0.05 (0.3%) | 0.02 (0.4%) |
| First-generation cephalosporins | 0.23 (1.3%) | 2.83 (52.9%) |
| Third-generation cephalosporins | 0.12 (0.7%) | 0.04 (0.8%) |
| Penicillins | 15.50 (90.7%) | 2.18 (40.7%) |
| Quinolones | 0.35 (2.1%) | 0.03 (0.6%) |
| Tetracyclines | 0.17 (1.0%) | 0.04 (0.7%) |
| Macrolides | 0 | 0.02 (0.4%) |
| Multiple classes | 0 | <0.01 (0.02%) |

^a^ mg/PCU, mg per active ingredient per population correction unit (% total PCU).
