# Supplementary figures and images for: Prevalence and distribution of extended-spectrum β-lactamase and AmpC-producing Escherichia coli in two New Zealand dairy farm environments
Source: Front Microbiol. 2022 Aug 11;13:960748. doi: 10.3389/fmicb.2022.960748 (PMC9403332; doi:10.3389/fmicb.2022.960748)

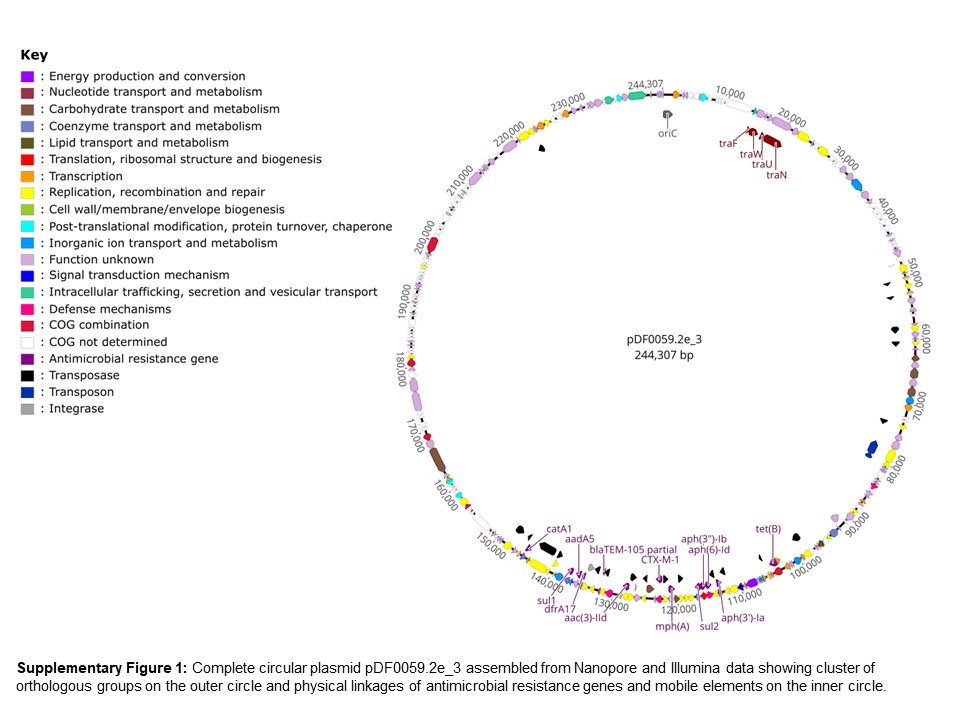

Supplement: Supplementary file 11 [file Image_1.jpg]

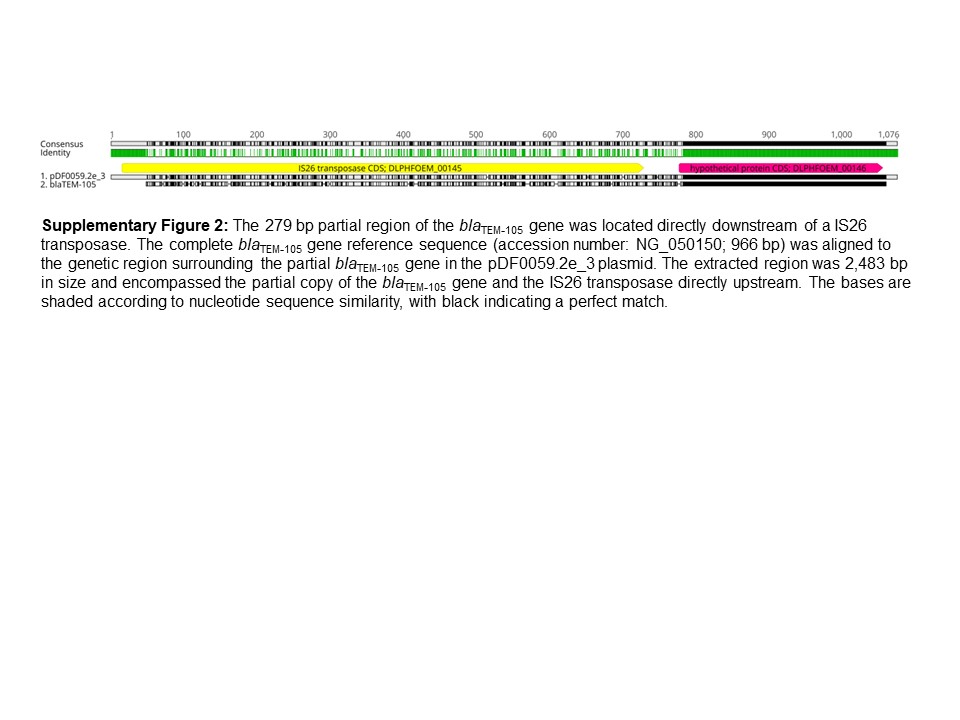

Supplement: Supplementary file 12 [file Image_2.jpg]

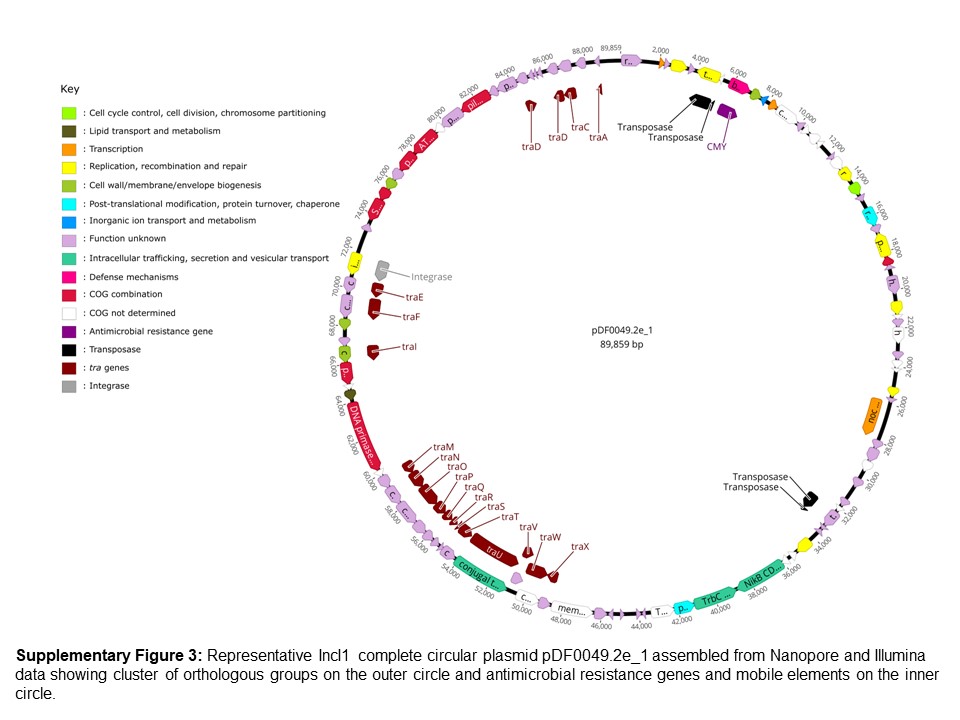

Supplement: Supplementary file 13 [file Image_3.jpg]
